# Combined analyses of transcriptome and metabolome reveal the mechanism of exogenous strigolactone regulating the response of elephant grass to drought stress

**DOI:** 10.3389/fpls.2023.1186718

**Published:** 2023-05-08

**Authors:** Jing Zhou, Yijia Liu, Yan Li, Wenqing Ling, Xiaoyu Fan, Qixian Feng, Ray Ming, Fulin Yang

**Affiliations:** ^1^ National Engineering Research Center of Juncao Technology, Fujian Agriculture and Forestry University, Fuzhou, China; ^2^ College of Animal Sciences (College of Bee Science), Fujian Agriculture and Forestry University, Fuzhou, China; ^3^ Center for Genomics and Biotechnology, Fujian Provincial Key Laboratory of Haixia Applied Plant Systems Biology, Fujian Agriculture and Forestry University, Fuzhou, China

**Keywords:** transcriptome, metabolome, exogenous strigolactone, drought resistance, SL crosstalk, elephant grass

## Abstract

Elephant grass is widely used in feed production and ecological restoration because of its huge biomass and low occurrence of diseases and insect pets. However, drought seriously affects growth and development of this grass. Strigolactone (SL), a small molecular phytohormone, reportedly participates in improving resilience to cope with arid environment. But the mechanism of SL regulating elephant grass to response to drought stress remains unknown and needs further investigation. We conducted RNA-seq experiments and identified 84,296 genes including 765 and 2325 upregulated differential expression genes (DEGs) and 622 and 1826 downregulated DEGs, compared drought rehydration with spraying SL in roots and leaves, respectively. Combined with targeted phytohormones metabolite analysis, five hormones including 6-BA, ABA, MeSA, NAA, and JA had significant changes under re-watering and spraying SL stages. Moreover, a total of 17 co-expression modules were identified, of which eight modules had the most significant correlation with all physiological indicators with weighted gene co-expression network analysis. The venn analysis revealed the common genes between Kyoto Encyclopedia of Genes and Genomes enriched functional DEGs and the top 30 hub genes of higher weights in eight modules, respectively. Finally, 44 DEGs had been identified as key genes which played a major role in SL response to drought stress. After verification of its expression level by qPCR, six key genes in elephant grass including *PpPEPCK*, *PpRuBPC*, *PpPGK*, *PpGAPDH*, *PpFBA*, and *PpSBPase* genes regulated photosynthetic capacity under the SL treatment to respond to drought stress. Meanwhile, *PpACAT*, *PpMFP2*, *PpAGT2*, *PpIVD*, *PpMCCA*, and *PpMCCB* regulated root development and phytohormone crosstalk to respond to water deficit conditions. Our research led to a more comprehensive understanding about exogenous SL that plays a role in elephant grass response to drought stress and revealed insights into the SL regulating molecular mechanism in plants to adapt to the arid environment.

## Introduction

Drought is one of the important environmental factors affecting plant growth and development. In recent years, with continuous changes in climate worldwide, the frequency and intensity of drought have been increasing (Banks et al., 2019). Severe drought may cause metabolic imbalance of plant cells, resulting in excess light energy and damage of photosynthetic organs. It may also cause the accumulation of reactive oxygen species in plant leaves, accelerate biofilm lipid peroxidation and inhibit plant growth. Plants have evolved various defense and self-protection mechanisms in the long-term evolution process to deal with the influence of drought stress. It could regulate the photosynthetic process of leaves, remove excess reactive oxygen species, and maintain certain turgor pressure in cells by closing stomata in leaves, activating the antioxidant system, and increasing osmotic substances, finally maintain sustainable growth of plants under drought stress (Yamada and Umehara, 2015; Muhammad et al., 2017).

In addition to their own stress response, exogenous growth regulators help improve the drought tolerance of plants. The common growth regulators include abscisic acid (ABA), gibberellin (GA), auxin, cytokinin, brassinosteroids, ethylene, and some small molecular metabolites discovered in recent years, such as nitric oxide (NO), jasmonic acid (JA), salicylic acid (SA), polyamines and strigolactones (SLs) (Koltai et al., 2010; Daviere and Achard, 2016). Plant growth regulators are generally recognized to promote stomatal closure, reduce transpiration rate, and decrease water loss under water-deficit growth environment (Yamada and Umehara, 2015). They could also delay the degradation rate of chlorophyll in leaves and lessen the damage degree of chloroplast caused by drought. In addition, they could enhance the enzymatic activity and increase the proline contents to reduce lipid peroxidation (Visentin et al., 2020). Therefore, growth regulators play an important role in plant response to drought stress.

SLs are a small class of carotenoid-derived compounds and they were first characterized as crystalline germination stimulants in root parasitic plants, including *Striga*, *Phelipanche*, and *Orobanche* species (Cook et al., 1966). Subsequently, SLs were found as a class of phytohormones with root-derived signal that could enhance the symbiosis relationship between plants and arbuscular mycorrhizal fungi, playing a remarkable role in the suppression of shoot branching and tillering (Chen et al., 2014). Meanwhile, studies have shown that SLs could not only affect the development and growth process of plants but also devote to the regulatory systems of plant stress adaptation for establishing multiple abiotic stress tolerances in plants (Bhoi et al., 2021). For example, spraying exogenous SL on arid *Vitis Vinifera* and wheat (*Triticum aestivum*) seedlings could reduce stomatal opening, electrolyte leakage and H_2_O_2_, reactive oxygen species, and malondialdehyde (MDA) content, to regulate its ability to cope with drought stress (Min et al., 2018).

A single phytohormone regulates several aspects of plant life cycle. Most developmental and growth processes are affected by various hormones simultaneously (de Saint Germain et al., 2013). The crosstalk of SLs with other phytohormone indicates that SLs accumulate in plant tissues to cope with different environmental conditions and actively contribute to multiple hormonal responsive pathways of plant stress adaptation (Bhoi et al., 2021). SLs positively regulate stress- and/or ABA-responsive gene expression in connection with plant development and abiotic stress responses (Ha et al., 2014; Haider et al., 2018). SL application reduced the levels of zeatin riboside and indoleacetic acid (Ghorbel et al., 2021) and increased the level of ABA in leaves and roots under drought stress (Min et al., 2018). Increasing amounts of endogenous SLs in less-branched varieties may have restrained the concentration of endogenous CKs, leading to the inhibition of axillary buds in rice (*Oryza sativa*) (Xu et al., 2015). Similar results have also been demonstrated in *Zantedeschia aethiopica*, where bud growth was activated by SL and CK interaction (Manandhar et al., 2018). In addition to the above crosstalk, SL and GA were involved in seed germination and shoot branching. GA application negatively and independently regulated SL biosynthesis in rice and *Lotus japonicus* (de Saint Germain et al., 2013). Therefore, SLs adjust multiple hormonal reaction pathways, which improve the capacity of plant development and coping with environmental stress.

The mechanism of SL responding to drought stress differ among various plant species. The α/β hydrolases DWARF14 (D14), which acts as receptors of SL, interact with the F-box protein MORE AXILLARY GROWTH 2 (MAX2) to target SUPPRESSOR OF MAX2 1 (SMAX1)-LIKE/D53 family members for degradation *via* the 26S proteasome (Yao et al., 2016; Yang et al., 2019). However, in *Arabidopsis*, the SMAX2 1-LIKE 6, 7, and 8 (SMXL 6, 7, and 8) and SMAX1-LIKE2 (SMXL2) act as negative regulators of water deficit resistance, and destruction of these *SMXL* genes in plants may lead to an original strategy to improve their drought resistance (Li et al., 2020; Feng et al., 2022). In a mutant of *Hordeum vulgare*, the *hvd14.d* gene is unusually sensitive to drought stress, may be due to the disruption of ABA signaling and metabolism pathways, leading to the drought-sensitive phenotype in the SL mutant. The correlation between SL signal and drought stress response is dependent on ABA level (Marzec et al., 2020). Under drought stress in tomato (*Solanum lycopersicum*), the level of SL in the roots decreased, and it was considerable related to the down-regulation of *SlCCD7* and *SlCCD8* genes in the roots (Ruiz-Lozano et al., 2015). In rice, *OsTB1* and SL were found to be involved in tillering inhibition under low water-deficit treatment, which is independent of the flowering pathway (Du et al., 2018). Comparative analysis of rice SL mutants (D3, D10, D17 and D27) showed that ABA content was positively correlated with the expression of β-carotene isomerase encoding gene of D27, and the homeostasis of ABA was maintained by regulating this gene to increase the drought tolerance of plants (Haider et al., 2018).

Elephant grass (*Pennisetum purpureum* Schum.) is an ideal gramineae C_4_ plant for sand fixation, wind prevention, and water-soil conservation, on the account of its large biomass and developed root system (Zhou et al., 2021). It has been widely planted for full-scale environmental management of regions with vulnerable ecology (Zhou et al., 2021). Meanwhile, arid environment affects the yield and constraints its large-scale plantation. For improvement of its performance and productivity in water deficit conditions, exogenous SL was applied to improve drought resistance. RNA sequencing (RNA-seq) and hormone-targeted metabolism analysis, combined with physiological indices, could comprehensively and quickly acquire the gene expression of a specific tissue or cell in a certain state. The findings will improve the understanding of the exogenous SL on regulating the mechanism of drought stress tolerance in C_4_ grasses and offer a theoretical basis for genetic improvement and breeding of elephant grass.

## Materials and methods

### Experimental materials

Elephant grass sprouts were collected from the plantation base in National Herbage Cultivar Evaluation Station. After growing healthily and producing nine leaves, they were subjected to stop irrigation for 10 days under room temperature (27°C) to obtain drought-stressed plants. The experiment included three groups: normal watering treatment, drought rehydration management, and foliar spraying SL handling. Water and 3 μmol/L GR 24 (Beijing Solarbio Science & Technology Co., Itd, China), which is a synthetic SL, were applied to the leaves to make the surface fully coated with liquid. After 72 h, the three groups of plants were tested.

### Physiological index detection

#### Determination of photosynthetic indices of elephant grass

In this study, the photosynthetic indices of elephant grass were surveyed using a CIRAS-3 portable photosynthesizer (PP-Systems Company Amesbury, MA01913, USA) to test the top third unfolded leaf of the plant. The photosynthetic indices were commanded under the conditions of which light intensity, air relative humidity, and CO_2_ concentration were 1200 μmol·(m^2^·s) ^−1^, 75%, and 380 μmol·mol^−1^, respectively. The net photosynthetic rate (Pn), intercellular CO_2_ concentration (Ci), transpiration rate (Tr), stomatal conductance (Gs), and water use efficiency (WUE) were measured in each treatment group. Then, the leaves were dark-adapted for 20 min before Chl fluorescence parameter was determined. The performance index on an absorption basis (PIabs) and the maximum photochemical efficiency (Fv/Fm) were determined using a plant efficiency analyzer. Three plants were surveyed for each treatment.

#### Measurement of physiological indices

Taking the roots and leaves as sample, the physiological indices, including peroxidase (POD), superoxide dismutase (SOD) activity, and proline (PRO), and MDA contents, were determined in terms of a previously reported method. Meanwhile, leaf samples were snap frozen and ground in liquid nitrogen for photosynthetic enzyme activity test. Then, 0.1 g of the sample was placed into a centrifuge tube and added with 1 mL of extraction solution. The mixture was homogenized in an ice bath and centrifuged at 8000 g and 4°C for 10 min. Ten microliters of supernatant were collected for the determination of photosynthetic key enzyme nicotinamide adenine dinucleotide phosphate-malic enzyme (NADP-ME), phosphoenolpyruvate carboxylase (PEPC), and pyruvate phosphate dikinase (PPDK) activities in accordance with the NADP-ME kit, PEPC kit, and PPDK kit purchased from Shanghai Preferred Biotechnology Co., China, respectively. Three plants were taken as repetition for each treatment.

#### Roots measurements

After the roots were dug out from the soil medium, they were gently washed with running water until the attachment of roots was totally cleaned, and then one of the main roots was randomly selected. Next, 10 cm-long roots were taken from the stem as samples for measurement. The samples were scanned for images, and the root morphology index, including root length (Len), surface area (SA), number of tips (NTips), volume (Vol), and average diameter (AvgD), was measured with WinRHIZO. Every treatment had three replications. After the above physiological indices of plants were detected, the leaves and roots of fresh elephant grass were collected and immediately fixed with liquid nitrogen to prepare for subsequent hormone metabolism and transcriptome analysis.

### Targeted metabolite analysis of plant hormone under exogenous SL treatment

#### Metabolite extraction

About 50 mg of samples of elephant grass, including leaves and roots, was respectively placed in a 1.5 mL Eppendorf tube for phytohormone metabolite testing, with three replicates per treatment. All samples were added with 1 mL of 50% acetonitrile aqueous solution and ground at 4°C for 6 min by freezing tissue grinding mill (Wonbio-96E, Shanghai, China). The mixture was shocked by ultrasonic for 30 min, stood at 4°C for 30 min, and centrifuged at 13,000 g at 4°Cfor 15 min. All supernatants were taken for purification, and the eluant was placed into 2 mL Eppendorf tube to blow nitrogen to dry. Finally, 50 μL of 30% acetonitrile aqueous solution was added into the tube, mixed together, and centrifuged at 13,000 g at 4°C for 15 min. The solution was the metabolite extraction, and it was transferred to sample vials for LC-MS/MS analysis.

#### Targeted metabolite analysis by LC-MS/MS

Before the phytohormone concentration of the samples were tested, a total of 22 kinds of standards were melted with methanol and mixed together to obtain the standard stock solution. Phytohormone metabolites were tested using an ultrahigh-performance liquid phase chromatograph mass spectrometer (UHPLC-Qtrap). The mobile phases were 0.01% formic acid in water (phase A) and 0.01% formic acid in acetonitrile (solvent B). The mass spectral conditions were positive and negative modes, curtain gas of 35 psi, medium collision gas, ion spray voltage floating of +5500/−4500, and source temperature of 550°C.

#### Data preprocessing

By using AB Sciex quantitative software, the default indices were used to automatically authenticate and integrate each ion fragment. With the mass spectral peak area and the concentration of appointed hormone as ordinates and abscissa, respectively, the standard curves of linear regression were drawn. The concentration of sample hormones was calculated by substituting the mass spectral peak area of the samples into the linear equation. Finally, the phytohormone content of the samples (ng/mg) was calculated as being equal to the concentration of samples hormone multiplied by the sample extraction volume and divided by sample weight.

### Characteristics of transcriptome in exogenous SL treated drought elephant grass

#### Total RNA extraction and sequencing library preparation for transcriptome

Total RNA was extracted from the elephant grass seedling and roots by using the TRIzol reagent (TransGen, Beijing, China) following the manufacturer’s protocol. RNA integrity was measured using an Agilent Bioanalyzer 2100 (Agilent Technologies, Santa Clara, CA, USA). The concentration and purity of RNA were tested using a NanoDrop 1000 spectrophotometer (Thermo Fisher Scientific, Wilmington, DE, USA). Only high-quality RNA sample was used to construct sequencing library.

The RNA-seq library was constructed with the Illumina TruSeq RNA sample preparation kit. mRNA was linked with poly-T oligo-attached magnetic beads and then fragmented by a fragmentation buffer. Then, double-stranded cDNA was composited using a SuperScript double-stranded cDNA synthesis kit with random hexamer primers. The composite cDNA was subjected to phosphorylation, A base addition, and end repair. Library fragments were singled out with cDNA target fragments of 300 bp and sequenced with the Illumina NovaSeq 6000 sequencer.

#### Sequence read mapping and assembly

The raw paired-end reads were trimmed and quality controlled by SeqPrep (https://github.com/jstjohn/SeqPrep) and Sickle (https://github.com/najoshi/sickle) with default parameters. The genome of *Cenchrus purpureus* was used as reference gene, and the clean reads were separately aligned to it with orientation mode by using HISAT2 (http://ccb.jhu.edu/software/hisat2/index.shtml) software. The mapped reads of each sample were assembled by StringTie (https://ccb.jhu.edu/software/stringtie) in a reference-based approach.

#### Gene functional annotation and differential expression analysis

All the assembled transcripts were analyzed by BLAST with six public databases, including Kyoto Encyclopedia of Genes and Genomes (KEGG) pathway database, Gene Ontology (GO) database, Pfam protein family (Pfam) database, non-redundant (NR) protein sequence, eukaryotic orthologous group (KOG) database, and manually annotated and reviewed protein sequence database (Swiss-Prot). The expression of each transcript was calculated in accordance with the transcripts-per-million-reads (TPM) method. After the read count data were standardized, the DESeq2 package analyses were adjusted *via* Benjamini–Hochberg method to determine the false discovery rate (FDR). Differentially expressed genes (DEGs) were determined with *q*-value ≤ 0.01 and |log_2_FC| > 2.

#### KEGG pathway analysis of DEGs

The KEGG database was used for KEGG enrichment analysis of DEGs during the three treatments of elephant grass to study the DEGs of SL regulating drought stress. By estimating the variation of gene length, the probability of different gene-enriched KEGG pathways could be calculated more accurately. The statistical concentration of DEGs was examined using KOBAS 2.0 web server, and a corrected *p*-value < 0.05 was considered to be remarkable abundant in KEGG.

#### Weighted gene co-expression network analysis in drought resistance of elephant grass

WGCNA was used to probe the relationship between genes and physiological indicators, and between genes and genes. The DEGs with TPM < 1 were removed, and the remaining DEGs were inputted into the WGCNA network. Pearson’s correlation matrix and network topology analysis were used to calculate the soft thresholding power and the gene correlation, respectively. Then, the adjacent relationship was converted to a topological overlap matrix. In standard WGCNA networks, the mergeCutHeight, power, and minModuleSize values were set to 0.3, 5, and 20, respectively. The networks were visualized using Cytoscape version 3.7.2.

### Quantitative RT-PCR validation

Quantitative RT-PCR analysis was conducted on a CFX Connect qPCR detection system to validate the accuracy of the RNA-seq results. Six randomly selected genes and 12 identified key genes were detected, with *PpACTB* as the reference gene (the primers are shown in **Supplementary Table 4** as supplementary data). The 2^−ΔΔCT^ method was used to calculate the relative expression levels of genes.

### Statistical analysis

Physiological data were statistically analyzed using SPSS 20.0. The significance of differences between every treatment was detected by Duncan’s multiple comparative and one-way ANOVA (*P* < 0.05).

### Availability of data and materials

All data generated or analyzed during this study are included in this published article. The RNA-Seq data and the datasets presented in this study can be found at the NCBI repository, accession number PRJNA928572, https://submit.ncbi.nlm.nih.gov/subs/sra/SUB12636465/overview.

## Results

### Physiological evaluation of elephant grass seedlings in response to SL treatments

**Figure 1:**
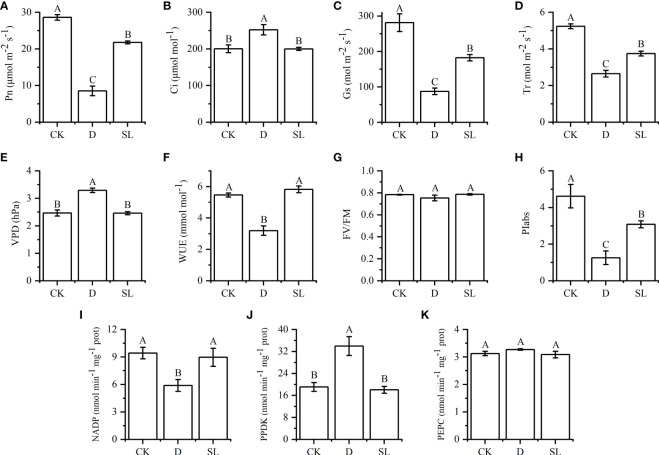
Effects of normal, drought-rehydration, and exogenous SL treatments on the growth of elephant grass **(A–K)** Photosynthesis-related indices, including Pn, net photosynthetic rate; Ci, intercellular CO_2_ concentration; Gs, stomatal conductance; Tr, transpiration rate; VPD, vapor pressure deficit; WUE, water use efficiency; FV/FM, optimal/maximal quantum yield of PSII; PIabs, photosynthetic performance index; NADP-ME, nicotinamide adenine dinucleotide phosphate-malic enzyme; PPDK, pyruvate phosphate dikinase; and PEPC, phosphoenolpyruvate carboxylase. Different capital letters in the same panel indicate statistical significance (*P* < 0.05).

Exogenous SL treatments affected the photosynthetic indices of elephant grass (**Figure 1**). Compared with normal watering treatment and drought rehydration management, the plants treated with SL showed a significant difference in Pn, Gs, Tr, and PIabs which were significantly higher than those in plants exposed to drought-rehydration condition and lower than those in plants exposed to normal watering environment. The values of Pn, Gs, Tr and PIabs were 21.77 μmol m^−2^ s^−1^, 182.67 mol m^−2^ s^−1^, 3.75 mol m^−2^ s^−1^, and 3.08, respectively (**Figures 1A, C, D, H**). The Ci, VPD, and WUE could be restored to CK under SL treatments, but in the drought-rehydration process, the values of Ci and VPD were higher than those in CK and SL treatment. Meanwhile, the values of WUE showed the opposite trend (**Figures 1B, E, F**). However, among all photosynthetic indices, Fv/Fm was not significantly affected by the three treatments, and the values were between 0.75 and 0.79 (**Figure 1G**).

For the key enzymes in photosynthetic reactions of leaves, NADP-ME and PPDK exhibited significant differences among the three treatments, especially after SL treatment. The values of NADP-ME and PPDK were 8.97 and 18.04 nmol^-1^ min^-1^ mg, respectively, which were similar to those under control condition. On the contrary, the values of NADP-ME and PPDK in drought-rehydration treatment were lower and higher than those in CK and SL treatments, respectively (**Figures 1I, G, K**).

**Figure 2:**
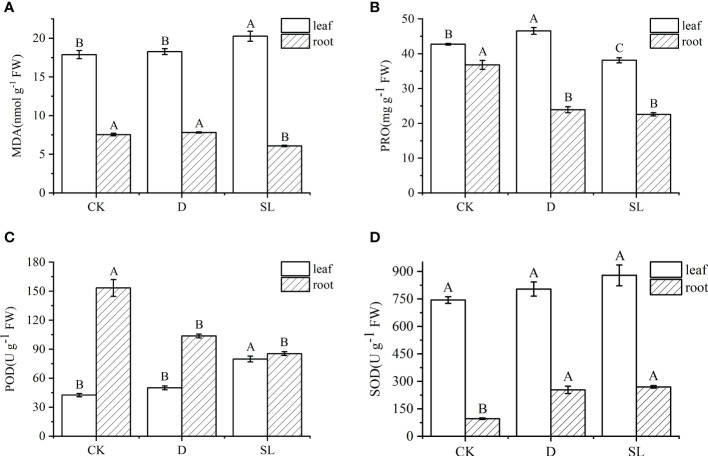
Physiological indices of elephant grass regulated under normal, drought-rehydration, and exogenous SL treatment conditions **(A)** Malondialdehyde (MDA), **(B)** proline (PRO), **(C)** peroxidase (POD), and **(D)** superoxide dismutase (SOD). Different capital letters in the same panel indicate statistical significance (*P* < 0.05).

After drought-rehydration and SL treatments, MDA, PRO, POD, and SOD showed different trends in the leaves and roots. The value of MDA in the leaves and roots under SL treatment were 20.27 and 6.08 nmol^-1^g FW, and both values in leaves and roots were significantly higher and lower than those under control and drought-rehydration conditions, respectively (**Figure 2A**). PRO and POD had the same decreased trend in the roots, the values of which were significantly less in drought-rehydration and SL treatment than in control conditions. Meanwhile, SOD had the opposite trend under the three different conditions with values significantly higher in drought-rehydration and SL treatments (**Figures 2B–D**). However, in leaves, the value of PRO and POD were 46.55 μg^-1^ g FW and 79.82 U g^-1^ FW under the drought-rehydration and SL treatment conditions, respectively (**Figures 2B, C**). SOD had no significant difference in leaves (**Figure 2D**).

**Figure 3:**
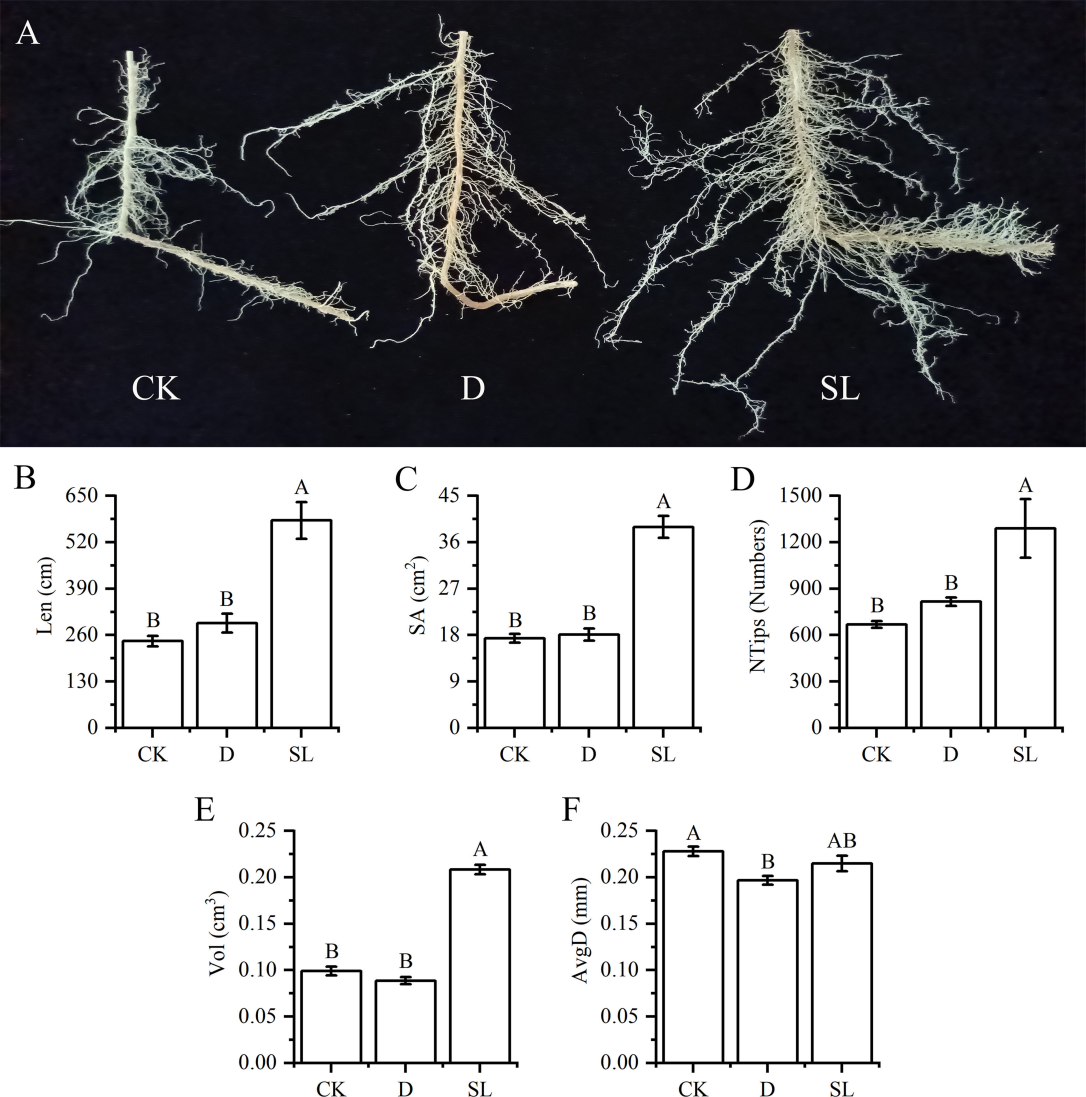
Growth of elephant grass roots under drought-rehydration and SL treatment compared with that under CK **(A)** Image of elephant grass roots under different treatment conditions. **(B–F)** Root length (Len), surface area (SA), number of tips (NTips), root volume (Vol) and average diameter (AvgD) among normal, drought-rehydration, and exogenous SL treatments of elephant grass. Different capital letters in the same panel indicate statistical significance (*P* < 0.05).

Different treatments created different growth conditions of elephant grass elephant grassroots. Spraying SL in elephant grass yielded more fibrous roots (**Figure 3A**). The values of Len, SA, NTips and Vol were 580.97 cm, 38.96 cm^3^, 1289.33, and 0.21 cm^3^ under SL treatment, respectively, which were significantly higher than those under control and drought-rehydration treatment (**Figures 3B–E**). Compared with normal growth condition, spraying SL had no significant difference and spraying water had remarkable discrepancy in the AvgD of roots, the values of which were 0.22 and 0.20 cm, respectively (**Figure 3F**).

**Figure 4:**
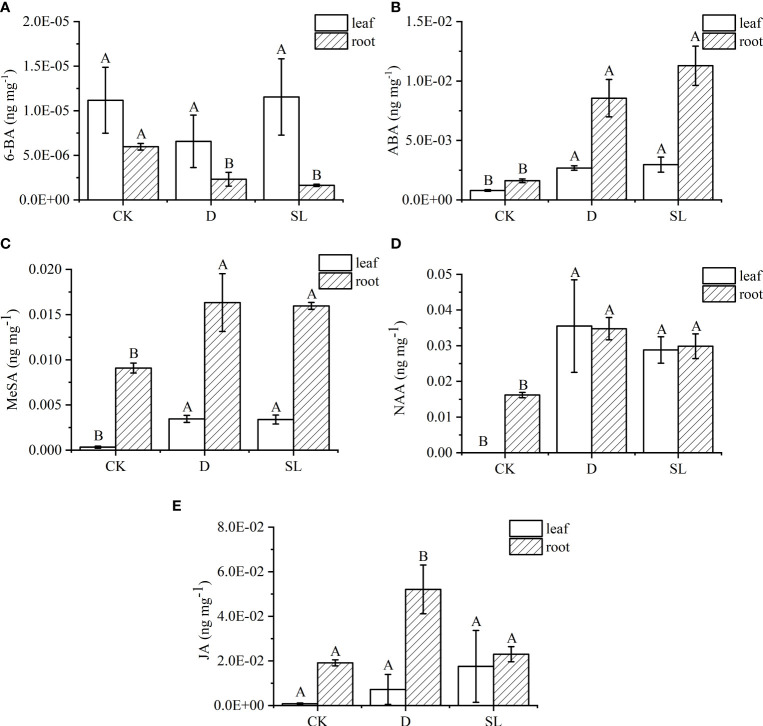
Hormone metabolite abundances among three different treatments of elephant grass on the basis of LC-MS analysis **(A–E)** Concentration of 6-benzylaminopurine (6-BA), abscisic acid (ABA), methyl salicylate (MeSA), naphthlcetic acid (NAA), and jasmonic acid (JA) between leaves and roots. Different capital letters in the same panel indicate statistical significance (*P* < 0.05).

A total of 22 hormones that are common and important in plants were further analyzed to accurately identify hormone metabolites affected by exogenous SL regulating the response of elephant grass to drought stress (**Supplementary File 1**). Only five hormones had significant difference in leaves and roots under different treatments. In roots, the concentration of 6-BA significantly decreased in drought-rehydration and SL treatments compared with that in control condition (**Figure 4A**). Meanwhile, the ABA, MeSA, and NAA concentrations had an opposite trend. In leaves, the values of ABA, MeSA, and NAA concentrations were 0.003, 0.003, and 0.029 ng^−1^ mg in SL treatment, respectively, higher than those in control condition (**Figures 4B–D**). The JA in leaves had no significant changes under spraying water and SL compared with that under CK (**Figure 4E**). JA was higher in roots under drought-rehydration treatment than under SL treatment at 0.05 ng^−1^ mg.

### Transcriptional analysis revealing key genes affecting SL regulation of elephant grass response to drought stress

A total of 18 RNA-seq libraries from CK, drought-rehydration, and SL treatments were constructed to explore the mechanism underlying SL regulating the response of elephant grass root and leaf to drought stress. All original FASTQ data files were submitted to the NCBI Sequence Read Archive under accession number SUB12636465. An overview of the sequencing is listed in **Supplementary Table 1**, and more than 98.3% and 95.0% of bases had *q*-value > 20 and 30 (an error probability of 0.02%), respectively. The GC-content ranged between 52.84% and 53.57% (**Supplementary Table 1**). After removing low-quality reads, a total of 797 clean reads were generated from the 18 samples, and 700 million mapped genes were compared with the reference genome (**Supplementary Table 1**). All 84,296 genes and 137,299 transcripts, including 57,491 known genes, 26,805 new genes, 53,975 known transcripts, and 83,324 new transcripts, were tested (**Supplementary Table 2**). The number of all known genes in GO, KEGG, KOG, NR, Swiss-Prot, and Pfam databases were 49,452 (86.0%), 25,627 (44.6%), 56,848 (98.9%), 57,400 (99.8%), 49,026 (85.3%), and 50,591 (88.0%), respectively (**Supplementary Table 3**).

**Figure 5:**
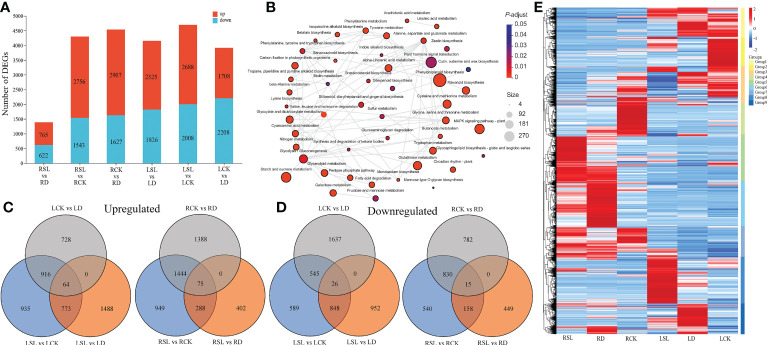
Analysis of all identified DEGs **(A)** Upregulated and downregulated DEGs by drought-rehydration and SL treatments in elephant grass. **(B)** Network of significantly enriched KEGG pathways using all detected DEGs. Colors correspond to groups of corrected *p*-value. **(C–D)** Venn diagrams showing the upregulated and downregulated DEGs across six comparisons. **(E)** Hierarchical clustering heatmap of all identified DEGs. Each column corresponds to control, drought-rehydration, and SL treatments in root and leaf, and nine groups were separated.

The gene expression levels were calculated and normalized using the TPM method; |log2FC| > 2 and *q*-value ≤ 0.01 were set for the screening of significant differential expression. In total, 765, 2,756, 2,907, 2,325, 2688 and 1,708 DEGs were upregulated, whereas 622, 1,543, 1,627, 1,826, 2,008 and 2,208 DEGs were downregulated at R1 (RSL vs. RD), R2 (RSL vs. RCK), R3 (RCK vs. RD), L1 (LSL vs. LD), L2 (LSL vs. LCK) and L3 (LCK vs. LD) combinations, respectively (**Figure 5A**). All the DEGs were annotated into the KEGG database, and 48 pathways were in the network, which had significant enrichment, including top three pathways (phenylpropanoid biosynthesis, nitrogen metabolism and alpha-Linolenic acid metabolism) (**Figure 5B**). All the upregulated and downregulated DEGs were shown at Venn figures, in which 1,488 and 402 upregulated DEGs were located at L1 and R1, respectively, and 589 and 540 downregulated DEGs were exhibited at the L2 and R2 combinations, respectively (**Figures 5C, D**). All the 13,656 identified DEGs were classified into nine groups by hierarchical clustering heatmap, and the genes in groups 5 and 8 had significantly high expression under SL treatment in the root and leaf, respectively (**Figure 5E**). The transcription factors (TFs) of 47 families were included in the DEGs during drought rehydration and spraying SL (**Supplementary File 2**). The largest three group of TFs was the basic helix-loop-helix family, myeloblastosis and ethylene responsive factor, which have 349, 345 and 292 TFs. Six genes were randomly selected to validate the accuracy of the RNA-seq results, and the relative expression levels of genes had the same tendency between the qPCR system and the RNA-seq results (**Supplementary Figure 1**).

### Generation of weighted gene co-expression network analysis module and hub gene analysis

**Figure 6:**
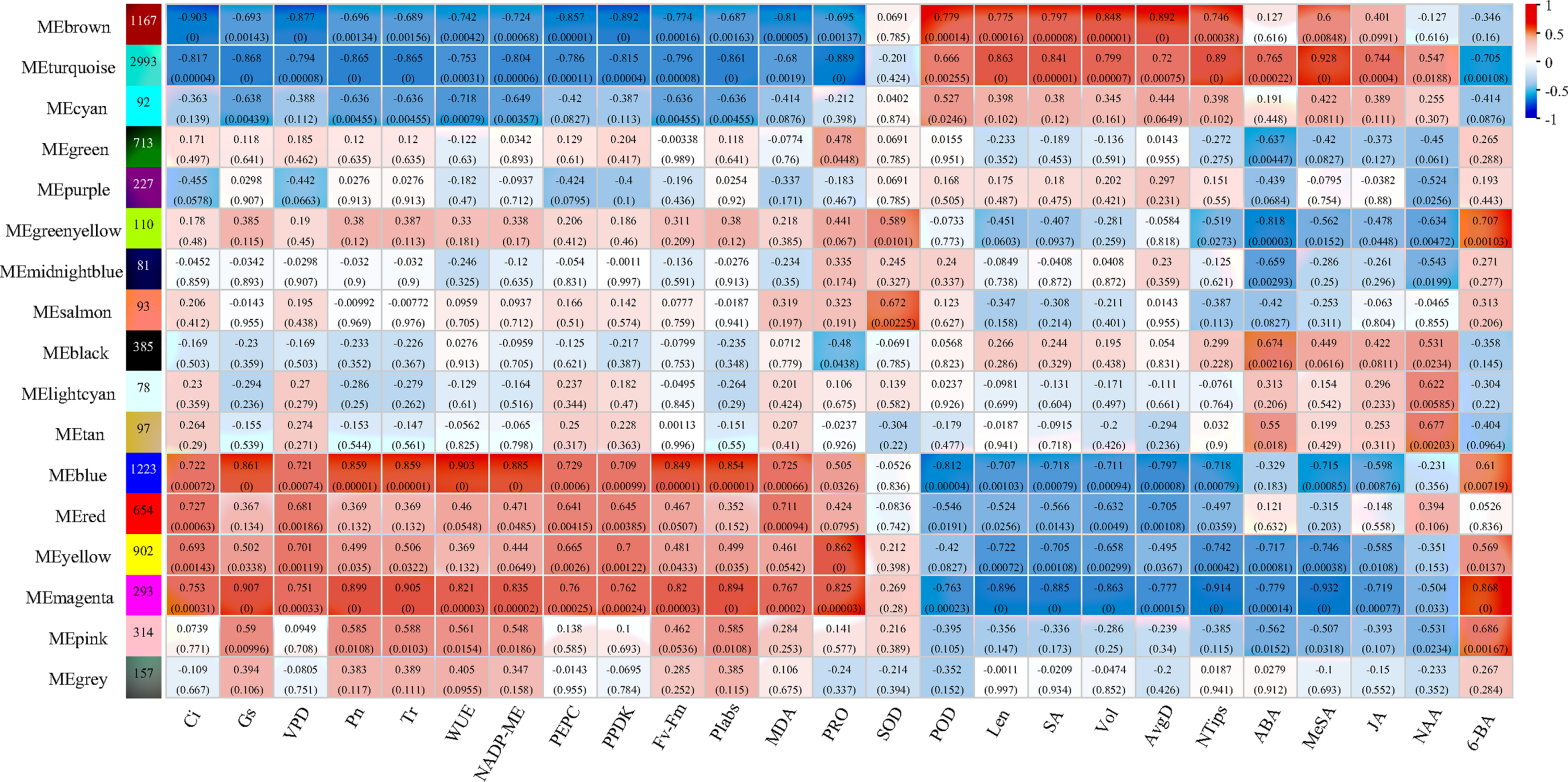
Module–trait relationship with physiological, root growth, and hormone indices of elephant grass The number represents the correlation coefficient of modules with physiological indices. The number in the bracket means *p*-value.

The gene co-expression profiles of elephant grass in response to control, drought-rehydration, and drought-SL treatments were analyzed using WGCNA to explore the relationship between genes and physiological indexes, especially genes in modules that were significantly correlated with physiological indicators. A total of 17 co-expression modules were identified and obtained, of which eight modules had the most significant correlation with all physiological indicators (**Figure 6**). The photosynthetic indices were negatively correlated with brown and turquoise modules and positively correlated with blue, magenta, and yellow module; the correlation coefficients were between -0.75 and -0.90 and between 0.75 and 0.91, respectively. However, the brown and turquoise modules had positive correlation with root conditions and phytohormone changes under different treatments, and the correlation coefficient was between 0.74 and 0.93.

**Figure 7:**
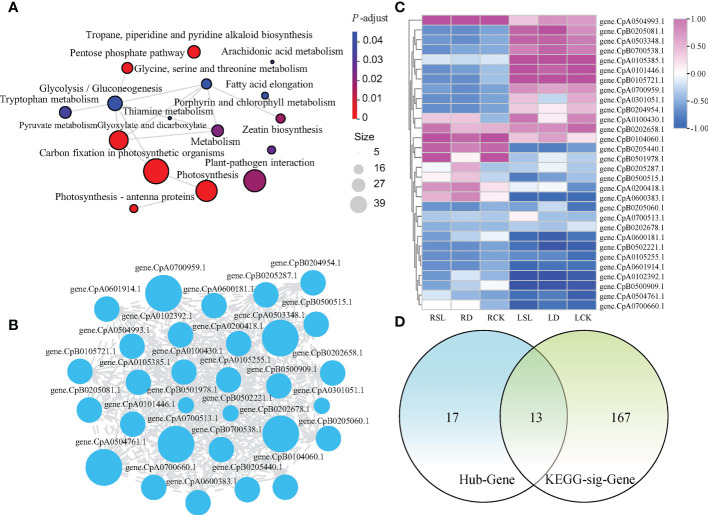
Analysis of 1223 genes in blue module **(A)** Network analysis of significant enrichment by KEGG pathway. **(B)** Cytoscape analysis of top 30 hub genes with a higher weight in blue module. **(C)** Hierarchical clustering heatmap of 30 hub genes. **(D)** Venn analysis revealing the common parts between KEGG enriched functional genes and the top 30 hub genes of higher weights in blue module.

A total of 1,223, 1,167, 2,993, 293, 97, 93, 902, and 110 genes could be found in the blue, brown, turquoise, magenta, tan, salmon, yellow and green-yellow modules, respectively (**Supplementary File 3**). Further analyses were conducted with the blue module as an example (**Figure 7**). Totally 1,223 genes were annotated in the KEGG pathway database, and 180 genes were significantly enriched in 16 pathways. The top three most significantly enriched pathways were carbon fixation in photosynthetic organisms, photosynthesis, and glyoxylate and dicarboxylate metabolism (**Figure 7A**). The top 30 hub genes with a higher weight in the blue module were selected for analysis and drawing the network. Genes *CpA0700513.1*, *CpB0202678.1*, *CpA0504761.1*, *CpA0503348.1*, and *CpA0700959.1* had higher degree than 517 (**Figure 7B**; **Supplementary File 4**). The TPM hierarchical clustering heatmap of 30 hub genes and the Venn analyses of 180 genes with KEGG function annotation were shown (**Figures 7C, D**). Among them, 13 genes were not only hub genes but also genes with significant enrichment function.

In accordance with the blue module analysis methods, 20, 22, two, five, five, 12, and three significantly enriched pathways and 202, 444, 8, 13, 11, 89, and 12 genes with KEGG enrichment function were identified on the other seven modules (**Supplementary Files 5**, **6**). The top 30 hub genes were shown as network and hierarchical clustering heatmap (**Supplementary File 7**; **Supplementary Figures 2**, **3**). The Venn analysis showed that the common parts of KEGG enrichment function genes and hub genes were five, two, one, eight, eight, zero, and seven genes in the brown, turquoise, magenta, tan, salmon, yellow, and green-yellow modules, respectively (**Supplementary Figure 4**). In general, in the eight modules that were significantly related to physiological indicators, 44 DEGs that played a major role in SL response to drought stress were identified (**Supplementary File 8**).

### Functional analysis and verification of key genes

For the 44 identified key DEGs, hierarchical clustering heatmaps were analyzed and the genes had more differential expression in leaves than in roots under exogenous SL regulating drought stress (**Supplementary Figure 5A**). The KEGG significant enrichment analysis network diagram of these genes showed that valine, leucine, and isoleucine degradation; carbon fixation in photosynthetic organisms; and fatty-acid degradation were the top three most significantly enriched pathways (**Supplementary Figure 5B**). Correlation coefficient analysis was performed to understand the relationship between the 44 key DEGs and various physiological indicators (**Supplementary Figure 5C**). Genes *CpA0101446.1*, *CpB0105721.1*, *CpB0104060.1*, and *CpB0700538.1* showed positive correlation with photosynthetic indices, physiology indicators, and one kind of phytohormone 6-BA and negative correlation with root growth and other phytohormones. In this analysis, the 44 identified DEGs became connected when the Network Analyst program was applied (**Supplementary Figure 5D**). Three independent groups, including purple, blue, and pink, were shown. In the purple group, many DEGs had high positive correlation coefficient with photosynthesis indices, whereas the DEGs in the pink group had negative correlation coefficient with phytohormone when SL was sprayed on elephant grass (**Supplementary Figures 5C, D**).

The FDR of 44 key DEGs under four comparisons was analyzed to further understand the response of exogenous SL treatment to drought in elephant grass (**Table 1**). Compared with drought-rehydration treatment, SL treatment showed many downregulated and upregulated genes located in the leaves. Phosphoenolpyruvate carboxykinase (*PpPEPCK*), ribulose bisphosphate carboxylase small chain A (*PpRuBPC*), phosphoglycerate kinase (*PpPGK*), glyceraldehyde-3-phosphate dehydrogenase A (*PpGADPH*), fructose-bisphosphate aldolase (*PpFBA*), and sedoheptulose-1,7-bisphosphatase (*PpSBPase*) had FDRs between 2.24 and 3.52. However, in roots, methylcrotonoyl-CoA carboxylase subunit alpha (*PpMCCA*), methylcrotonoyl-CoA carboxylase beta chain (*PpMCCB*), alanine-glyoxylate aminotransferase 2 homolog 1 (*PpAGT2*), isovaleryl-CoA dehydrogenase (*PpIVD*), acetyl-CoA acetyltransferase (*PpACAT*), and glyoxysomal fatty acid beta-oxidation multifunctional protein MFP-a (*PpMFP2*) were the most important DEGs, with FDRs of 4.12, 3.87, 2.66, 3.4, 3.82, 2.91, −2.34, −3.33, and −2.03, respectively.

**Table 1:** Analyzed direction of gene expression changes of 44 key DEGs involved in the eight mode during drought-rehydration and spraying SL of elephant grass.

	Direction of gene expression changes					
Gene ID	LCK vs LSL	LSL vs LD	RCK vs RSL	RSL vs RD	Gene description	Abbreviation
gene.Cp0000455.1			4.12		Methylcrotonoyl-CoA carboxylase subunit alpha	*MCCA*
gene.CpA0101446.1		3.55			Phosphoenolpyruvate carboxykinase	*PEPCK*
gene.CpA0102214.1			2.66		Methylcrotonoyl-CoA carboxylase beta chain	*MCCB*
gene.CpA0103469.1	-2.51		-2.26		Probable glycerol-3-phosphate dehydrogenase	*GPDH*
gene.CpA0104386.1					Acyl-coenzyme A oxidase 4	*ACOX4*
gene.CpA0203545.1					5’-nucleotidase SurE	*surE*
gene.CpA0301051.1		3.52			Ribulose bisphosphate carboxylase	*RuBPC*
gene.CpA0403417.1	-2.52		-2.54		3-ketoacyl-CoA thiolase 2	*KAT2*
gene.CpA0504761.1	-2.4	2.16			Photosystem I chlorophyll a/b-binding protein 5	*CAB5*
gene.CpA0504993.1		2.03			Aspartate aminotransferase	*AST*
gene.CpA0700959.1	-2.04	2.84			Sedoheptulose-1,7-bisphosphatase	*SBPase*
gene.CpA0702908.1			2.4		2-oxoacid dehydrogenases acyltransferase	*BLAODA*
gene.CpA0703361.1			-2.34		Acetyl-CoA acetyltransferase	*ACAT*
gene.CpB0103289.1			-3.33		Glyoxysomal fatty acid beta-oxidation multifunctional protein MFP-a	*MFP2*
gene.CpB0103297.1			-2.03		Glyoxysomal fatty acid beta-oxidation multifunctional protein MFP-a	*MFP2*
gene.CpB0104060.1	-2.36	2.24			Phosphoglycerate kinase	*PGK*
gene.CpB0105196.1			2.91		Isovaleryl-CoA dehydrogenase	*IVD*
gene.CpB0105721.1		2.53			Fructose-bisphosphate aldolase	*FBA*
gene.CpB0203332.1	4.12				Chlorophyll a-b binding protein 2	*CAB2*
gene.CpB0205081.1	-2.64				Photosystem II 22 kDa protein 1	*PSBS1*
gene.CpB0500657.1			3.82		Alanine-glyoxylate aminotransferase 2 homolog 1	*AGT2*
gene.CpB0700538.1		2.29			Glyceraldehyde-3-phosphate dehydrogenase A	*GAPDH*
gene.CpA0100504.1			2.1		Xylanase inhibitor protein 1	*XIP1*
gene.CpA0102576.1			2.57		Quinolinate synthase	*QS*
gene.CpA0104511.1					Isovaleryl-CoA dehydrogenase	*IVD*
gene.CpA0105385.1		2.75			Fructose-bisphosphate aldolase	*FBA*
gene.CpA0201611.1			-2.2		Phospholipase D delta	*PLDdelta*
gene.CpA0201626.1			-2.15		Phospholipase D delta	*PLDdelta*
gene.CpA0202256.1	-2.77		-3.77		flavonoid 3’-monooxygenase	*F3’M*
gene.CpA0202265.1	-3.9		-3.11		Trimethyl tridecatetraene synthase	*TMTT*
gene.CpA0203312.1			2.15		Primary amine oxidase 2	*PrAO2*
gene.CpA0301066.1			3.17		Probable O-methyltransferase 2	*OMT2*
gene.CpA0303976.1			3.87		Methylcrotonoyl-CoA carboxylase subunit alpha	*PpMCCA*
gene.CpA0503348.1	-2.43	2.71			Glyceraldehyde-3-phosphate dehydrogenase A	*GAPDH*
gene.CpA0600615.1			2.07		Alcohol dehydrogenase-like 5	*ADH5*
gene.CpB0102025.1			3.4		Methylcrotonoyl-CoA carboxylase beta chain	*MCCB*
gene.CpB0103652.1			2.1		Peroxidase 15	*POD15*
gene.CpB0201645.1			2.66		2-oxoacid dehydrogenases acyltransferase	*BLAODA*
gene.CpB0204000.1			-2.58		Mitogen-activated protein kinase kinase kinase 17	*MAPKKK17*
gene.CpB0204954.1		2.86			Sedoheptulose-1,7-bisphosphatase	*SBPase*
gene.CpB0205060.1	-2.53				Photosystem II 22 kDa protein 1	*PSBS1*
gene.CpB0301112.1					Probable uridine nucleosidase 2	*URH2*
gene.CpB0400578.1			3.25	2.29	Indole-3-acetaldehyde oxidase	*IAAId*
gene.CpB0700954.1	-3.8		-2.19		Beta-glucosidase 18	*BGLU18*

red, upregulated DEGs.

green, downregulated DEGs.

The qPCR results of core genes were observed, and they proved the correctness of gene expression. The results showed that *PpPEPCK*, *PpRuBPC*, *PpPGK*, *PpGADPH*, *PpFBA*, and *PpSBPase* had higher expression in SL treatment than in normal and drought-rehydration treatments in leaves (**Supplementary Figure 6**). In roots, *PpACAT* and *PpMFP2* had low expression under SL treatment, whereas *PpAGT2*, *PpIVD*, *PpMCCA*, and *PpMCCB* had the opposite expression under spraying SL (**Supplementary Figure 6**).

## Discussion

SL is a recently discovered class of carotenoid-derived phytohormones and has multiple biological functions, from regulating plant architecture and stimulating the germination of parasitic plant seeds to participating in a range of plant development processes, including root growth, leaf senescence, and photomorphogenesis. In addition, SL could be associated with various stress environments, and it supports a positive potential strategy for abiotic stress tolerance in plants (Ha et al., 2014; Li et al., 2019). In this study, a comprehensive approach was taken to reveal the effect of physiological response during drought-rehydration and SL recovery stages in elephant grass seedlings. Combined analyses of transcriptome and metabolome were applied to investigate the response and molecular mechanism of exogenous SL on C_4_ plant under drought stress.

### Response of photosynthesis to SL regulating drought stress in elephant grass

Foliar application of SL on elephant grass largely ameliorated drought stress symptoms. In particular, photosynthesis had different photosynthetic capacities during the three growth conditions. The recovery ability after re-watering is usually considered important for successful adaptation of plants to arid conditions. Rehydration helps plants offset the damage and recover their physiological functions from drought stress (Zhou et al., 2021). Comparison of different recovering conditions with water and SL demonstrated that spraying SL provided elephant grass with better recovery capacity, with Pn, Gs, Tr, WUE, and PIabs being significantly higher than those under drought-rehydration condition (**Figures 1A, C, D, F, H**). These five indices are a comprehensive reflection of the photosynthetic and WUE characteristics of plants. The alleviation of drought damage by SL was significantly better than that by rewatering treatment, indicating that SL was more effective in promoting plant recovery under drought stress. SL could improve the photosynthetic capacity of plants, and it showed higher tolerance to drought stress (Li et al., 2023). This result is similar to that in other species, such as *V. vinifera* (Min et al., 2018; Li et al., 2021), maize seedlings (Sattar et al., 2022), and winter wheat (*Triticum aestivum*) (Sedaghat et al., 2021). Therefore, SL has stronger ability to repair drought plants than that of only using water.

Elephant grass as a model C_4_ plant species has higher photosynthesis due to a repertoire of C_4_ enzymes, including NADP-ME, PPDK, and PEPC, to enhance CO_2_ fixation (Li et al., 2022). The activities of the three enzymes above could improve photosynthesis efficiency, enhance stress tolerance, and ultimately increase crop yield under adverse conditions (Chen et al., 2019; He et al., 2020). PPDK and PEPC are the key enzymes that convert CO_2_ in the atmosphere into oxaloacetic acid (OAA) after being absorbed through stomata in mesophyll cells, while NADP-ME is a crucial enzyme that concentrates CO_2_ in bundle sheath cells (Huang et al., 2016). NADP-ME and PPDK had the opposite variation trend under drought-rehydration and SL treatments, possibly because after spraying SL under drought stress, the Gs of plants was more open than that under drought-rehydration conditions. Therefore, the absorbed CO_2_ content was higher, which reduced the activity of PPDK that catalyzes the conversion of CO_2_ to OAA. This phenomenon leads to a decrease in intercellular CO_2_ concentration and finally promotes an increase in the activity of NADP-ME in the CO_2_ concentration mechanism to improve the photosynthetic capacity. This result also showed that different treatment methods could cause diverse enzyme activities at distinct stages, thus leading to different photosynthetic capacity, which is a strategy for plants to adapt to changed environments.

### Insights into the root system and SL crosstalk profiling with other phytohormones

The root system is the main organ for nutrient and water absorption in plants, even those faced with severe drought environment. However, water deficit conditions usually lead to self-thinning of the root system, abscise some death roots and decreased root-absorption capacity (Kim et al., 2020; Li et al., 2021). SL is generally considered to inhibit adventitious root formation and regulate the elongation of primary roots in eudicot plants while increasing the number of adventitious roots and promoting the elongation of seminal roots in grass plants in short time (Sun et al., 2022). This opinion also confirmed the results of the present study that SL treatment could significantly increase the Len, SA, NTips, and Vol of elephant grass. Similar results were found in *A. thaliana*, that is, SL positively controls root elongation and regulates the primary root length by regulating the content of *A. thaliana* growth hormone (Ruyter-Spira et al., 2011). The exogenous application of SL increases the length of the original roots, improves root phenotype, and promotes growth and development under stress conditions.

The results exhibited a complex phytohormonal response coordinated from multiple signaling pathways to respond to drought, in which ABA, MeSA, and NAA showed seemingly same increasing trends in roots and leaves under stress, whereas 6-BA showed a decreased tendency in roots. ABA is considered a “stress hormone,” whose levels could be constantly adjusted to adapt to environmental conditions (Liu et al., 2022). The ABA–SL interaction has been studied from various perspectives effectively, and inconsistencies in the interaction were found, which could be caused by the crosstalk with other phytohormones that could have antagonism on two components (Kaniganti et al., 2022). The expression levels of SL biosynthetic genes go down in ABA-deficient lines and SL induced mir156 accumulation, which increased guard cell sensitivity to ABA and subsequently generates stomatal closure for decreased water loss (Brun, 2020; Kaniganti et al., 2022). In consequence, the network regulation of SL is still inseparable from ABA.

SL has been proposed to serve as a modulator of auxin transport to regulate root elongation (Ruyter-Spira et al., 2011; Hu et al., 2018), and some research found that plant may increase SL production to promote symbiosis establishment to respond to stress (Ruiz-Lozano et al., 2015). In tall fescue, SL positively promoted crown root elongation under abiotic stress due to the interference of NAA transport and regulation of cell division (Du et al., 2018). Meanwhile, exogenous application of NAA was incapable of restoring root-hair elongation and symmetric root growth in the presence of SL on tomato (Koltai et al., 2010). However, in the present research, the content of NAA in leaves and roots was significantly higher than that in the control in elephant grass, may be due to the indirect correlation between SL and NAA during the interaction of plant growth regulators. Moreover, the increase in NAA content is more obviously related to the external environment.

Overall, the SL crosstalk with other phytohormones is complicated, and it needs further exploration. In this research, the SL contents and SL synthetic pathway were not identified using LC-MS/MS and RNA-Seq in roots and leaves, respectively. First, elephant grass may not produce endogenous SL by themselves. Second, as a signal molecule, SL may be quickly degraded and absorbed in plants through phytohormone metabolism.

### Key genes regulating SL response of elephant grass to drought stress

**Figure 8:**
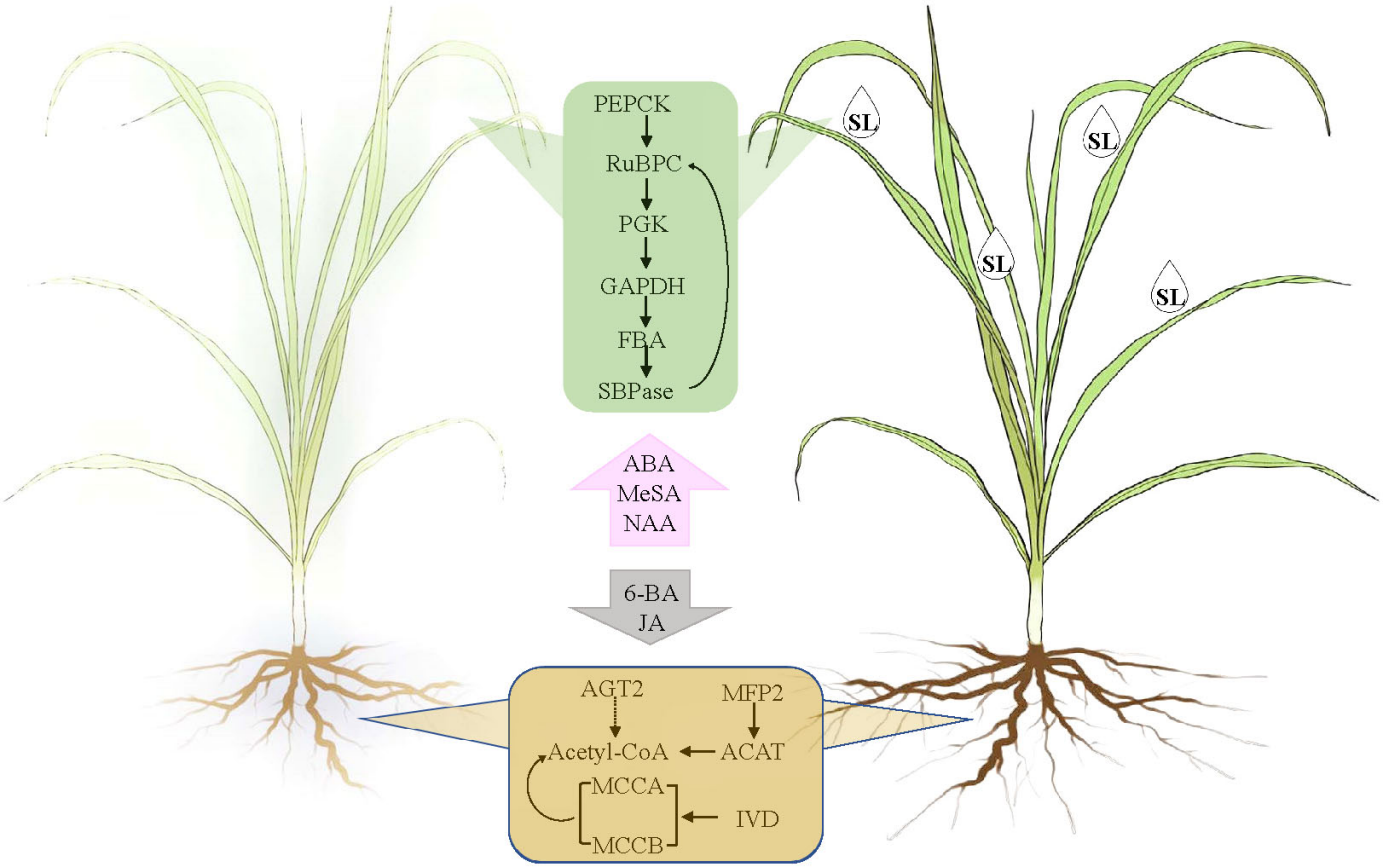
Model of the key hormones and gene adjustment of drought root and leaf elephant grass in response to SL treatment.

According to the WGCNA and correlation analyses, 18 unigenes encoding 12 genes were identified as the key genes for SL regulating the response of elephant grass to drought stress. Among them, *PpPEPCK*, *PpRuBPC*, *PpPGK*, *PpGAPDH*, *PpFBA*, and *PpSBPase* were demonstrated to be profusely expressed in the leaves, whereas *PpACAT*, *PpMFP2*, *PpAGT2*, *PpIVD*, *PpMCCA*, and *PpMCCB* were abundantly expressed in the roots of elephant grass. A model showing SL affects elephant grass to cope with drought stress from the leaves to the roots was proposed (**Figure 8**).

SL regulated the response of elephant grass to drought stress from carbon fixation in photosynthetic organisms; valine, leucine and isoleucine degradation; and fatty acid degradation pathways. PEPCK plays a very important role in plant metabolism, and it is involved in regulating gluconeogenesis and catalyzing the transformation of fat or malic acid into sugar. It could also participate in photosynthesis as a key CO_2_ concentrating enzyme in C_4_ plants (Lee et al., 2022). PEPCK catalyzed the reversible decarboxylation of OAA to form PEP and CO_2_, which increased the concentration of CO_2_ in the sheath cells of vascular bundles and saturated the concentration of CO_2_ in Rubisco substrate, thus improving the efficiency of photosynthesis (Huang et al., 2016).

Meanwhile, GAPDH catalyzes the reduction of 3-phosphoglycerate to triose phosphate, a key step in carbon reduction phase of photosynthesis. Both enzymes have been researched in Kentucky bluegrass, and the results exhibited that under drought stress and re-watering recovery conditions, carbon reduction regulated by GAPDH and carboxylation controlled by Rubisco could be the key metabolic processes of genetic variation in Pn responses to drought stress, and active Rubisco and GAPDH could be involved in superior post-drought recovery (Xu et al., 2013). In plants, PGK not only catalyzes the reversible conversion of 1,3-bisphosphoglycerate to 3-phosphoglycerate in glycolysis but also participates in gluconeogenesis and the Calvin-Benson cycle (Rosa-Tellez et al., 2018; Massange-Sánchez et al., 2020). GAPDH and PGK participate in the same compartment, and at the same time, in photosynthetic and glycolytic reactions (Rosa-Tellez et al., 2018). These key genes showed similar performance during the process of plant recovery and the gene expression increased sharply, which also reflected the main regulatory mechanism of SL in the process of regulating the growth of elephant grass.

*PpACAT* and *PpMFP2* were downregulated in the roots under SL treatment. These genes influence acetyl-CoA, which, as the core enzyme, is the connection site of valine, leucine, and isoleucine degradation and fatty-acid degradation pathways based on the KEGG enrichment analysis. Meanwhile, *PpIVD*, *PpMCCA* and *PpMCCB* were focused on fatty-acid metabolism, and they were upregulated in the roots under SL treatment. Combination of root physiological indices showed that SL treatment considerably improved the growth of roots and promoted the development of fibrous roots. This finding may be the result of the interaction regulation of root development by phytohormone interaction. However, how the identified genes worked on root growth must still be explored.

## Data availability statement

The datasets presented in this study can be found in online repositories. The names of the repository/repositories and accession number(s) can be found in the article/**Supplementary Material**, This data can be found here: https://dataview.ncbi.nlm.nih.gov/object/PRJNA928572.

## Author contributions

JZ conceived and performed the experiments and wrote the manuscript. YJL, YL, WL, XF, and QF performed parts of the experiments. RM supported the ideas of experiment and modified the manuscript. FY designed the experiments, provided experimental place, and financial assistance. All authors contributed to the article and approved the submitted version.

## Supplementary material

Supplementary File 1 A total of 22 hormones metabolites.

Supplementary File 2 TF analysis results.

Supplementary File 3 The genes identified in the eight key modules.

Supplementary File 4 The degree of top 30 hub genes with a higher weight in eight modules.

Supplementary File 5 KEGG pathways significant enrichment in eight modules.

Supplementary File 6 The genes in the KEGG significant enrichment pathway in eight modules.

Supplementary File 7 The top 30 hub genes in eight modules.

Supplementary File 8 The key 44 genes.

## Glossary

**Table:**

SL	Strigolactone
Pn	Net photosynthetic rate
Gs	Stomatal conductance
Tr	Transpiration rate
Ci	Intercellular CO2 concentration
WUE	Water use efciency
Fv/Fm	Maximum photochemical efciency
PIabs	Performance index based on absorbed light energy
PEPC	Phosphoenolpyruvate carboxylase
PPDK	Pyruvate phosphate dikinase
NADP-ME	NADP-malic enzyme
PEPCK	Phosphoenolpyruvate carboxykinase
ABA	Abscisic acid
6-BA	6-Benzylaminopurine
MeSA	methyl salicylate
NAA	naphthlcetic acid
JA	jasmonic acid
Len	Root length
SA	surface area
NTips	number of tips
Vol	root volume
AvgD	average diameter
RuBPC	Ribulose bisphosphate carboxylase
PGK	Phosphoglycerate kinase
FBA	Fructose-bisphosphate aldolase
GAPDH	Glyceraldehyde-3-phosphate dehydrogenase A
SBPase	Sedoheptulose-1
7-bisphosphatase	
MCCB	Methylcrotonoyl-CoA carboxylase beta chain
MCCA	Methylcrotonoyl-CoA carboxylase subunit alpha
MFP2	Glyoxysomal fatty acid beta-oxidation multifunctional protein MFP-a
IVD	Isovaleryl-CoA dehydrogenase
ACAT	Acetyl-CoA acetyltransferase
AGT2	Alanine-glyoxylate aminotransferase

## References

[B1] Banks J. M. Percival G. C. Rose G. (2019). Variations in seasonal drought tolerance rankings. Trees-Struct Funct. 33 (4), 1063–1072. doi: 10.1007/s00468-019-01842-5

[B2] Bhoi A. Yadu B. Chandra J. Keshavkant S. (2021). Contribution of strigolactone in plant physiology, hormonal interaction and abiotic stresses. Planta 254 (2), 28. doi: 10.1007/s00425-021-03678-1 34241703

[B3] Brun G. (2020). At The crossroads of strigolactones and abscisic acid pathways: a role for miR156. Plant Cell Environ. 43 (7), 1609–1612. doi: 10.1111/pce.13787 32406550

[B4] Chen M. MacGregor D. R. Dave A. Florance H. Moore K. Paszkiewicz K.. (2014). Maternal temperature history activates flowering locus T in fruits to control progeny dormancy according to time of year. Proc. Natl. Acad. Sci. U.S.A. 111 (52), 18787–18792. doi: 10.1073/pnas.1412274111 25516986 PMC4284563

[B5] Chen Q. Q. Wang B. P. Ding H. Y. Zhang J. Li S. C. (2019). Review: the role of NADP-malic enzyme in plants under stress. Plant Sci. 281, 206–212. doi: 10.1016/j.plantsci.2019.01.010 30824053

[B6] Cook C. E. Whichard L. P. Turner B. Wall M. E. Egley G. H. (1966). Germination of witchweed (Striga lutea lour.): isolation and properties of a potent stimulant. Science 154 (3753), 1189–1190. doi: 10.1126/science.154.3753.1189 17780042

[B7] Daviere J. M. Achard P. (2016). A pivotal role of DELLAs in regulating multiple hormone signals. Mol. Plant 9 (1), 10–20. doi: 10.1016/j.molp.2015.09.011 26415696

[B8] de Saint Germain A. Ligerot Y. Dun E. A. Pillot J. P. Ross J. J. Beveridge C. A.. (2013). Strigolactones stimulate internode elongation independently of gibberellins. Plant Physiol. 163 (2), 1012–1025. doi: 10.1104/pp.113.220541 23943865 PMC3793021

[B9] Du H. Huang F. Wu N. Li X. H. Hu H. H. Xiong L. H. (2018). Integrative regulation of drought escape through ABA-dependent and-independent pathways in rice. Mol. Plant 11 (4), 584–597. doi: 10.1016/j.molp.2018.01.004 29366830

[B10] Feng Z. Liang X. Tian H. Yasuko W. Huu N. K. Duy T. C.. (2022). Suppressor of MAX2 1 (SMAX1) and SMAX1-LIKE2 (SMXL2) negatively regulate drought resistance in arabidopsis thaliana. Plant Cell Physiol. 63 (12), 1900–1913. doi: 10.1093/pcp/pcac080 35681253

[B11] Ghorbel M. Zribi I. Missaoui K. Drira-Fakhfekh M. Azzouzi B. Brini F. (2021). Differential regulation of the durum wheat pathogenesis-related protein (PR1) by calmodulin TdCaM1.3 protein. Mol. Biol. Rep. 48 (1), 347–362. doi: 10.1007/s11033-020-06053-7 33313970

[B12] Ha C. V. Leyva-Gonzalez M. A. Osakabe Y. Tran U. T. Nishiyama R. Watanabe Y.. (2014). Positive regulatory role of strigolactone in plant responses to drought and salt stress. Proc. Natl. Acad. Sci. U.S.A. 111 (2), 851–856. doi: 10.1073/pnas.1322135111 24379380 PMC3896162

[B13] Haider I. Andreo-Jimenez B. Bruno M. Bimbo A. Floková K. Abuauf H.. (2018). The interaction of strigolactones with abscisic acid during the drought response in rice. J. Exp. Bot. 69 (9), 2403–2414. doi: 10.1093/jxb/ery089 29538660

[B14] He Y. F. Xie Y. F. Li X. Yang J. (2020). Drought tolerance of transgenic rice overexpressing maize C4-PEPC gene related to increased anthocyanin synthesis regulated by sucrose and calcium. Biol. Plant 64, 136–149. doi: 10.32615/bp.2020.031

[B15] Hu Q. N. Zhang S. X. Huang B. R. (2018). Strigolactones and interaction with auxin regulating root elongation in tall fescue under different temperature regimes. Plant Sci. 271, 34–39. doi: 10.1016/j.plantsci.2018.03.008 29650155

[B16] Huang C. F. Chang Y. M. Lin J. J. Yu C. P. Lin H. H. Liu W. Y.. (2016). Insights into the regulation of c-4 leaf development from comparative transcriptomic analysis. Curr. Opin. Plant Biol. 30, 1–10. doi: 10.1016/j.pbi.2015.12.011 26828378

[B17] Kaniganti S. Bhattacharya J. Petla B. P. Reddy P. S. (2022). Strigolactone, a neglected plant hormone, with a great potential for crop improvement: crosstalk with other plant hormones. Environ. Exp. Bot. 204, 105072. doi: 10.1016/j.envexpbot.2022.105072

[B18] Kim Y. Chung Y. S. Lee E. Tripathi P. Heo S. Kim K. H. (2020). Root response to drought stress in rice (Oryza sativa l.). Int. J. Mol. Sci. 21 (4), 1513. doi: 10.3390/ijms21041513 32098434 PMC7073213

[B19] Koltai H. Dor E. Hershenhorn J. Joel D. M. Weininger S. Lekalla S.. (2010). Strigolactones’ effect on root growth and root-hair elongation may be mediated by auxin-efflux carriers. J. Plant Growth Regul. 29 (2), 129–136. doi: 10.1007/s00344-009-9122-7

[B20] Lee M. S. Boyd R. A. Ort D. R. (2022). The photosynthetic response of c-3 and c-4 bioenergy grass species to fluctuating light. Glob. Chang. Biol. 14 (1), 37–53. doi: 10.1111/gcbb.12899

[B21] Li C. Dong L. Durairaj J. Guan J. C. Yoshimura M. Quinodoz P.. (2023). Maize resistance to witchweed through changes in strigolactone biosynthesis. Science 379 (6627), 94–99. doi: 10.1126/science.abq4775 36603079

[B22] Li W. Q. Herrera-Estrella L. Tran L. S. P. (2019). Do cytokinins and strigolactones crosstalk during drought adaptation? Trends Plant Sci. 24 (8), 669–672. doi: 10.1016/j.tplants.2019.06.007 31277931

[B23] Li Y. Li S. T. Feng Q. X. Zhang J. Han X. L. Zhang L.. (2022). Effects of exogenous strigolactone on the physiological and ecological characteristics of pennisetum purpureum schum. seedlings under drought stress. BMC Plant Biol. 22 (1), 578. doi: 10.1186/s12870-022-03978-y 36510126 PMC9743734

[B24] Li C. N. Li L. Reynolds M. P. Wang J. Y. Chang X. P. Mao X. G.. (2021). Recognizing the hidden half in wheat: root system attributes associated with drought tolerance. J. Exp. Bot. 72 (14), 5117–5133. doi: 10.1093/jxb/erab124 33783492

[B25] Li W. Q. Nguyen K. H. Tran C. D. Watanabe Y. Tian C. J. Yin X. J.. (2020). Negative roles of strigolactone-related SMXL6, 7 and 8 proteins in drought resistance in arabidopsis. Biomolecules 10 (4), 607. doi: 10.3390/biom10040607 32295207 PMC7226073

[B26] Liu J. Shu D. F. Tan Z. L. Ma M. Guo N. Gao S.. (2022). The arabidopsis IDD14 transcription factor interacts with bZIP-type ABFs/AREBs and cooperatively regulates ABA-mediated drought tolerance. New Phytol. 236 (3), 929–942. doi: 10.1111/nph.18381 35842794

[B27] Manandhar S. Funnell K. A. Woolley D. J. Cooney J. M. (2018). Interaction between strigolactone and cytokinin on axillary and adventitious bud development in zantedeschia. Physiol. Mol. Biol. Plants 6 (1), 1–6. doi: 10.4172/2329-955x.1000172

[B28] Marzec M. Daszkowska-Golec A. Collin A. Melzer M. Eggert K. Szarejko I. (2020). Barley strigolactone signalling mutant hvd14.d reveals the role of strigolactones in abscisic acid-dependent response to drought. Plant Cell Environ. 43 (9), 2239–2253. doi: 10.1111/pce.13815 32501539

[B29] Massange-Sánchez J. A. Casados-Vázquez L. E. Juarez-Colunga S. Sawers R. J. H. Tiessen A. (2020). The phosphoglycerate kinase (PGK) gene family of maize (Zea mays var. B73). Plants (Basel) 9 (12), 1639. doi: 10.3390/plants9121639 33255472 PMC7761438

[B30] Min Z. Li R. Y. Chen L. Zhang Y. Li Z. Y. Liu M.. (2018). Alleviation of drought stress in grapevine by foliar-applied strigolactones. Plant Physiol. Biochem. 135, 99–110. doi: 10.1016/j.plaphy.2018.11.037 30529172

[B31] Muhammad N. Muhammad S. N. Rashid A. Z.I. M. Y.A. M. Yasir H.. (2017). Foliar calcium spray confers drought stress tolerance in maize *via* modulation of plant growth, water relations, proline content and hydrogen peroxide activity. Arch. Agron. Soil Sci. 64 (1), 1–1. doi: 10.1080/03650340.2017.1341108

[B32] Rosa-Tellez S. Djoro Anoman A. Flores-Tornero M. Toujani W. Alseek S. Fernie A. R.. (2018). Phosphoglycerate kinases are Co-regulated to adjust metabolism and to optimize growth. Plant Physiol. 176 (2), 1182–1198. doi: 10.1104/pp.17.01227 28951489 PMC5813584

[B33] Ruiz-Lozano J. M. Aroca R. Zamarreno A. M. Molina S. Andreo-Jiménez B. Porcel R.. (2015). Arbuscular mycorrhizal symbiosis induces strigolactone biosynthesis under drought and improves drought tolerance in lettuce and tomato. Plant Cell Environ. 39 (2), 441–452. doi: 10.1111/pce.12631 26305264

[B34] Ruyter-Spira C. Kohlen W. Charnikhova T. Zeijl A. V. Bezouwen L. V. Ruijter N. D.. (2011). Physiological effects of the synthetic strigolactone analog GR24 on root system architecture in arabidopsis: another belowground role for strigolactones? Plant Physiol. 155 (2), 721–734. doi: 10.1104/pp.110.166645 21119044 PMC3032462

[B35] Sattar A. Ul-Allah S. Ijaz M. Sher A. Butt M. Abbas T.. (2022). Exogenous application of strigolactone alleviates drought stress in maize seedlings by regulating the physiological and antioxidants defense mechanisms. Cereal Res. Commun. 50 (2), 263–272. doi: 10.1007/s42976-021-00171-z

[B36] Sedaghat M. Emam Y. Mokhtassi-Bidgoli A. Hazrati S. Lovisolo C. Visentin I.. (2021). The potential of the synthetic strigolactone analogue GR24 for the maintenance of photosynthesis and yield in winter wheat under drought: investigations on the mechanisms of action and delivery modes. Plants (Basel) 10 (6), 1223. doi: 10.3390/plants10061223 34208497 PMC8233996

[B37] Sun H. W. Li W. Q. Burritt D. J. Tian H. T. Zhang H. Liang X. H.. (2022). Strigolactones interact with other phytohormones to modulate plant root growth and development. Crop J. 10 (6), 1517–1527. doi: 10.1016/j.cj.2022.07.014

[B38] Visentin I. Pagliarani C. Deva E. Caracci A. Tureková V. Novák O.. (2020). A novel strigolactone-miR156 module controls stomatal behaviour during drought recovery. Plant Cell Environ. 43 (7), 1613–1624. doi: 10.1111/pce.13758 32196123

[B39] Xu L. X. Yu J. J. Han L. B. Huang B. R. (2013). Photosynthetic enzyme activities and gene expression associated with drought tolerance and post-drought recovery in Kentucky bluegrass. Environ. Exp. Bot. 89, 28–35. doi: 10.1016/j.envexpbot.2012.12.001

[B40] Xu J. X. Zha M. R. Li Y. Ding Y. F. Chen L. Ding C. Q.. (2015). The interaction between nitrogen availability and auxin, cytokinin, and strigolactone in the control of shoot branching in rice (Oryza sativa l.). Plant Cell Rep. 34 (9), 1647–1662. doi: 10.1007/s00299-015-1815-8 26024762

[B41] Yamada Y. Umehara M. (2015). Possible roles of strigolactones during leaf senescence. Plants (Basel) 4 (3), 664–677. doi: 10.3390/plants4030664 27135345 PMC4844400

[B42] Yang T. Lian Y. Wang C. (2019). Comparing and contrasting the multiple roles of butenolide plant growth regulators: strigolactones and karrikins in plant development and adaptation to abiotic stresses. Int. J. Mol. Sci. 20 (24), 6270. doi: 10.3390/ijms20246270 31842355 PMC6941112

[B43] Yao R. F. Ming Z. H. Yan L. M. Li S. H. Wang F. Ma S.. (2016). DWARF14 is a non-canonical hormone receptor for strigolactone. Nature 536 (7617), 469–473. doi: 10.1038/nature19073 27479325

[B44] Zhou J. Chen S. Q. Shi W. J. David-Schwartz R. Li S. T. Yang F. L.. (2021). Transcriptome profiling reveals the effects of drought tolerance in giant juncao. BMC Plant Biol. 21 (1), 2. doi: 10.1186/s12870-020-02785-7 33390157 PMC7780708

