# Supplementary material for: Combined analyses of transcriptome and metabolome reveal the mechanism of exogenous strigolactone regulating the response of elephant grass to drought stress
Source: Front Plant Sci. 2023 May 8;14:1186718. doi: 10.3389/fpls.2023.1186718 (PMC10200884; doi:10.3389/fpls.2023.1186718)
Supplement: Supplementary file 9 [file Table_1.docx]

**Supplementary Table 1 Overview of the sequencing and the results of clean reads compared with reference genome**

|  | Samples | Raw reads | Raw bases | Clean reads | Clean bases | Error rate (%) | | Q20(%) | Q30(%) | GC contents (%) | Total mapped | Multiple mapped | Uniquely mapped |
| --- | --- | --- | --- | --- | --- | --- | --- | --- | --- | --- | --- | --- | --- |
|  | CK | 44660811 | 6743782511 | 44073653 | 6438050000 | | 0.02 | 98.37 | 95.21 | 53.57 | 39153020 | 3185301 | 35967719 |
| Leaf | DR | 49171720 | 7424929720 | 48649675 | 7132720612 | | 0.02 | 98.35 | 95.14 | 53.28 | 43915771 | 3618755 | 40297015 |
|  | SL | 43082602 | 6505472902 | 42639806 | 6313988815 | | 0.02 | 98.33 | 95.06 | 53.25 | 37832353 | 3104445 | 34727907 |
|  | CK | 43826647 | 6617823747 | 43239886 | 6376204976 | | 0.02 | 98.27 | 94.93 | 53.14 | 36782965 | 2900793 | 33882171 |
| Root | DR | 45494177 | 6869620777 | 44884164 | 6567418458 | | 0.02 | 98.25 | 94.90 | 52.96 | 39191645 | 3082968 | 36108677 |
|  | SL | 42951787 | 6485719787 | 42288833 | 6212290468 | | 0.02 | 98.31 | 95.03 | 52.84 | 36367646 | 2938186 | 33429460 |
