# Supplementary material for: Combined analyses of transcriptome and metabolome reveal the mechanism of exogenous strigolactone regulating the response of elephant grass to drought stress
Source: Front Plant Sci. 2023 May 8;14:1186718. doi: 10.3389/fpls.2023.1186718 (PMC10200884; doi:10.3389/fpls.2023.1186718)
Supplement: Supplementary file 10 [file Table_2.docx]

**Supplementary Table 2 All the expression analysis**

|  | All gene | Known gene | New gene | All transcript | Known transcript | New transcript |
| --- | --- | --- | --- | --- | --- | --- |
| number | 84296 | 57491 | 26805 | 137299 | 53975 | 83324 |
|  |  |  |  |  |  |  |
