# Supplementary material for: Combined analyses of transcriptome and metabolome reveal the mechanism of exogenous strigolactone regulating the response of elephant grass to drought stress
Source: Front Plant Sci. 2023 May 8;14:1186718. doi: 10.3389/fpls.2023.1186718 (PMC10200884; doi:10.3389/fpls.2023.1186718)
Supplement: Supplementary file 11 [file Table_3.docx]

**Supplementary Table 3 Annotation information of reference genes and transcripts in different databases**

| Database | Gene | | Transcript | |
| --- | --- | --- | --- | --- |
|  | number | percentage | number | percentage |
| GO | 49452 | 86.0% | 46326 | 85.8% |
| KEGG | 25627 | 44.6% | 23733 | 44.0% |
| KOG | 56848 | 98.9% | 53370 | 98.9% |
| NR | 57400 | 99.8% | 53894 | 99.9% |
| Swiss-Prot | 49026 | 85.3% | 45889 | 85.0% |
| Pfam | 50591 | 88.0% | 47477 | 88.0% |
| Total_annotation | 57418 | 99.9% | 53911 | 99.9% |
| Total | 57491 | 100% | 53975 | 100% |

Note: Gene number means number of genes in this project is annotated into the database; Transcript number means number of transcripts in this project is annotated into the database; All Gene number means number of genes in the species including new coding genes is annotated into the database; All Transcript number means number of transcripts in the species including new coding genes is annotated into the database.
