# Supplementary material for: Combined analyses of transcriptome and metabolome reveal the mechanism of exogenous strigolactone regulating the response of elephant grass to drought stress
Source: Front Plant Sci. 2023 May 8;14:1186718. doi: 10.3389/fpls.2023.1186718 (PMC10200884; doi:10.3389/fpls.2023.1186718)
Supplement: Supplementary file 12 [file Table_4.docx]

**Supplementary Table 4 Primers of 6 random gene and 12 key gene**

| Gene ID | Forward primer (5′-3′) | Reverse primer (5′-3′) |
| --- | --- | --- |
| gene.CpA0102972.1 | CAAGTGGCAGTTATCCGTGAT | CATCTGTGTGAGGTCGTTTGT |
| gene.CpA0300045.1 | GCCTTCTCTGACGACATTGAC | GTAGTAGAGCCTTCCACCTTGA |
| gene.CpA0704015.1 | GAACTGGCATAGCAGAACTCAT | GGACTGGACAGCATCAATCAA |
| gene.CpB0301345.1 | CCTGTTAGTGTCGCCTTCCA | TGAGTTCTTGATGAGCCAGTAGG |
| gene.CpB0401417.1 | TTCGGCTTCGTCACCTTCTC | TCATTGCGGAACAGTCTGGAA |
| gene.CpB0503145.1 | GCTCTTCACCTCCACCATCAT | ACGATCTTGGCCTCCATCAC |
| *PEPCK* | TTTCGGTTCCGCTTCGTCCTC | CCCGTTCTCCTGCTTCTTCTCC |
| *RBCS* | CACCTCTGTGGCTCCATTCC | CGAACTTCTTGTTGCCCTCC |
| *PGK* | GGCTGTTGCGAAGAAGTTGG | ACCTCCCGTTGAAATGTGGC |
| *GAPDH* | CTTCGTGAACGCCGTCTCCTT | GCTCCCGCCTGGATGTGCTT |
| *FBA* | CCTTCCCAAGAAGGCGGAAT | CCCTTGTCGACCTTGATCCC |
| *SBPase* | TGGGTGGTCCAGTTGAAGGTG | TAACGTAGGTCGTGCGAGGG |
| *ACAT* | CGTCCTCAGTGCCAACCTCG | CCTTCAGCATCCCATCAACC |
| *MFP2* | CATTGCTCTGGGTGGTGGAT | ACACGAGGCCCAGAGACTAT |
| *AGT2* | CCCGTCGCTCTTCCACTACTA | CATTGCTAACTCATTCGCTTC |
| *IVD* | CGCATCGTCCTCCCTCCTCT | ACATAGCCACCGTCCACTTT |
| *MCCA* | TTCTGGCTGCTGCATGTATT | CTATGTTGGGAGCTATCGTCA |
| *MCCB* | TAAAGGGCTCCCACTTCATT | GGATGCCACCCATAACAGAT |
