# Supplementary figures and images for: Combined analyses of transcriptome and metabolome reveal the mechanism of exogenous strigolactone regulating the response of elephant grass to drought stress
Source: Front Plant Sci. 2023 May 8;14:1186718. doi: 10.3389/fpls.2023.1186718 (PMC10200884; doi:10.3389/fpls.2023.1186718)

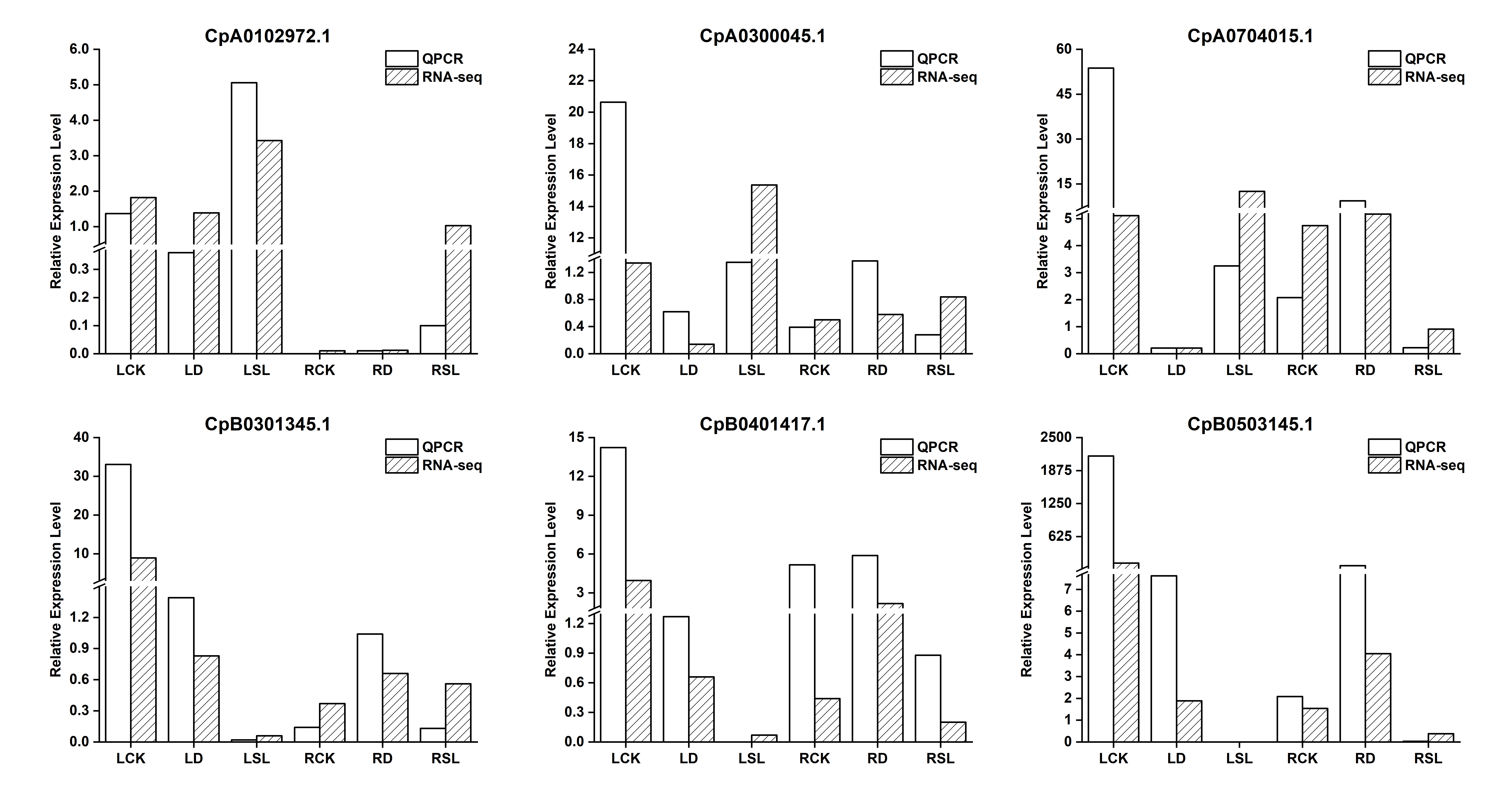

Supplement: Supplementary file 13 [file Image_1.tif]

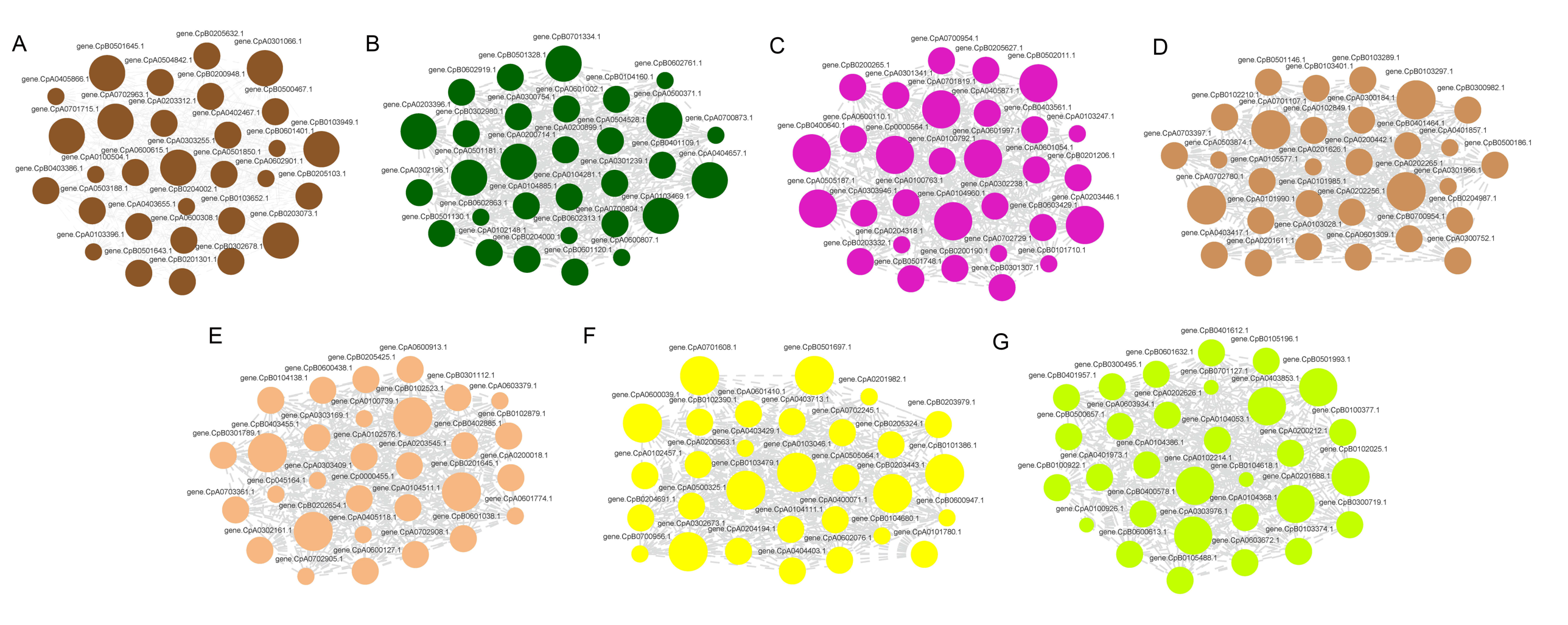

Supplement: Supplementary file 14 [file Image_2.tif]

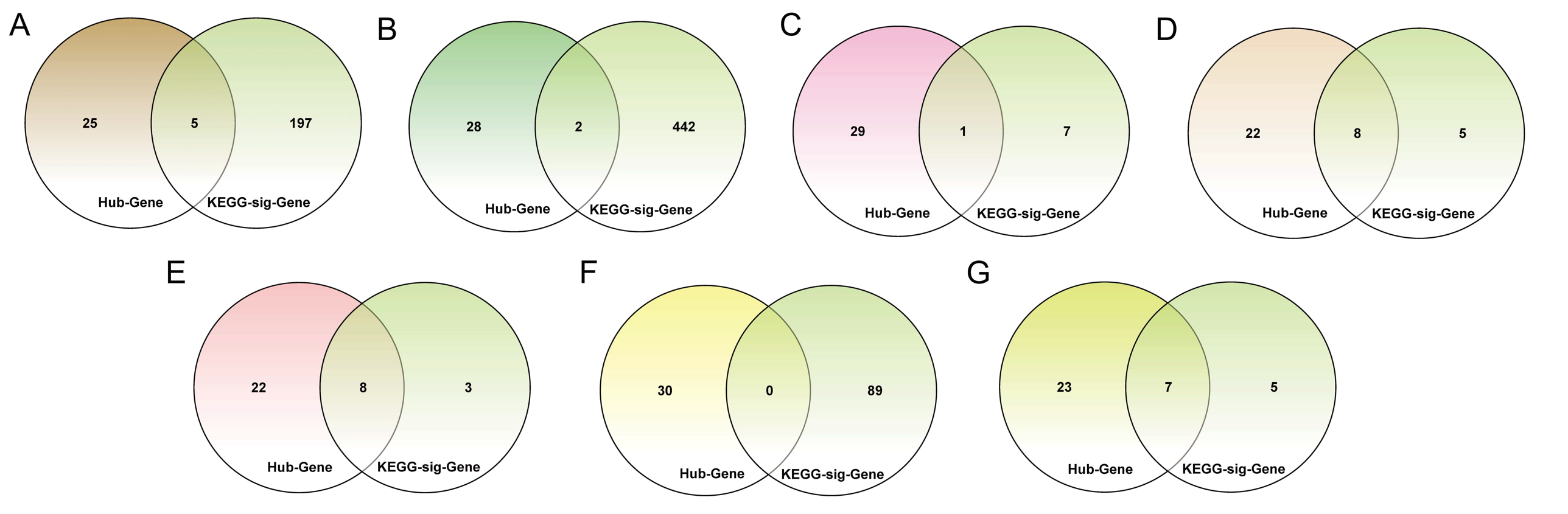

Supplement: Supplementary file 16 [file Image_4.tif]

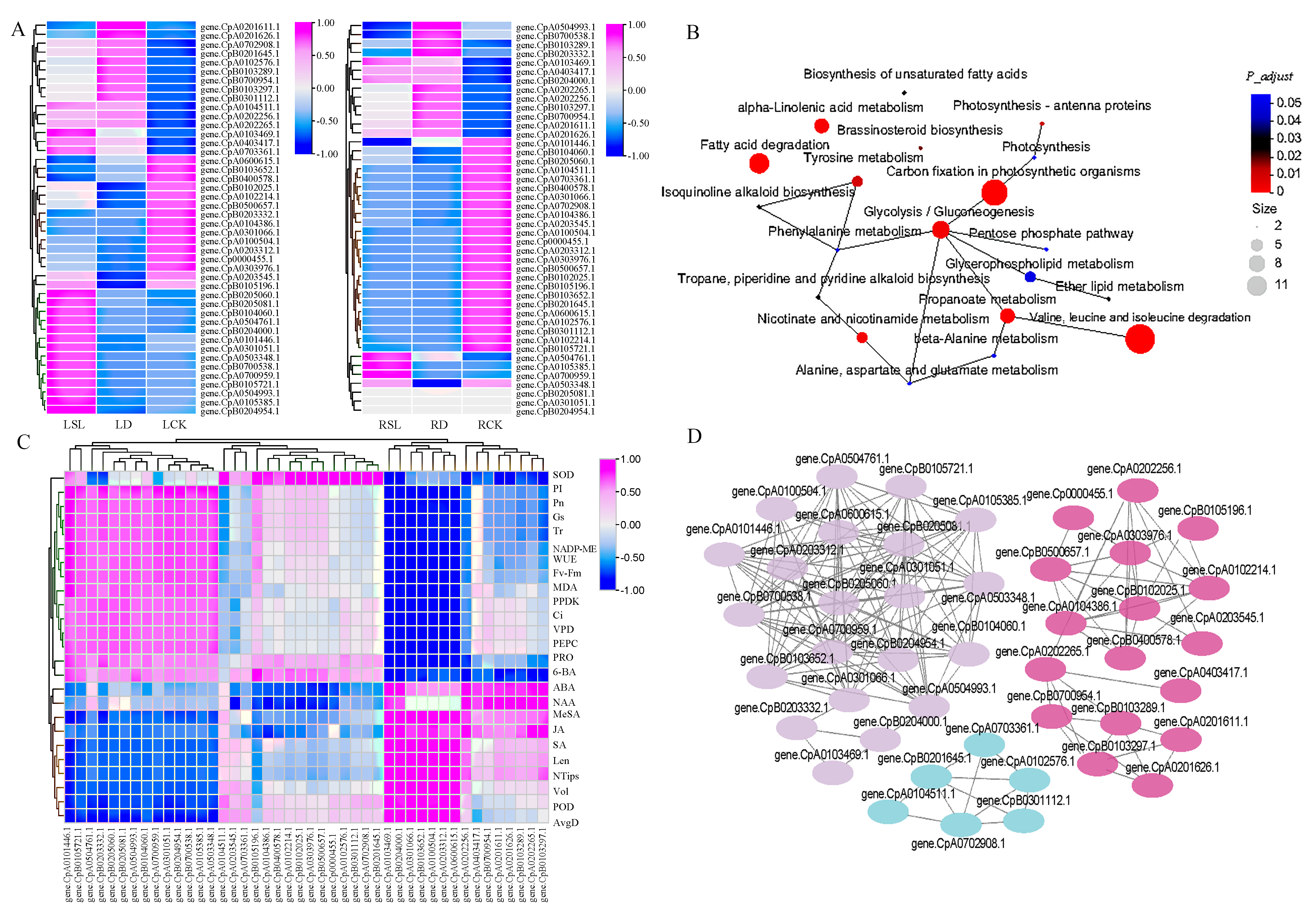

Supplement: Supplementary file 17 [file Image_5.tif]

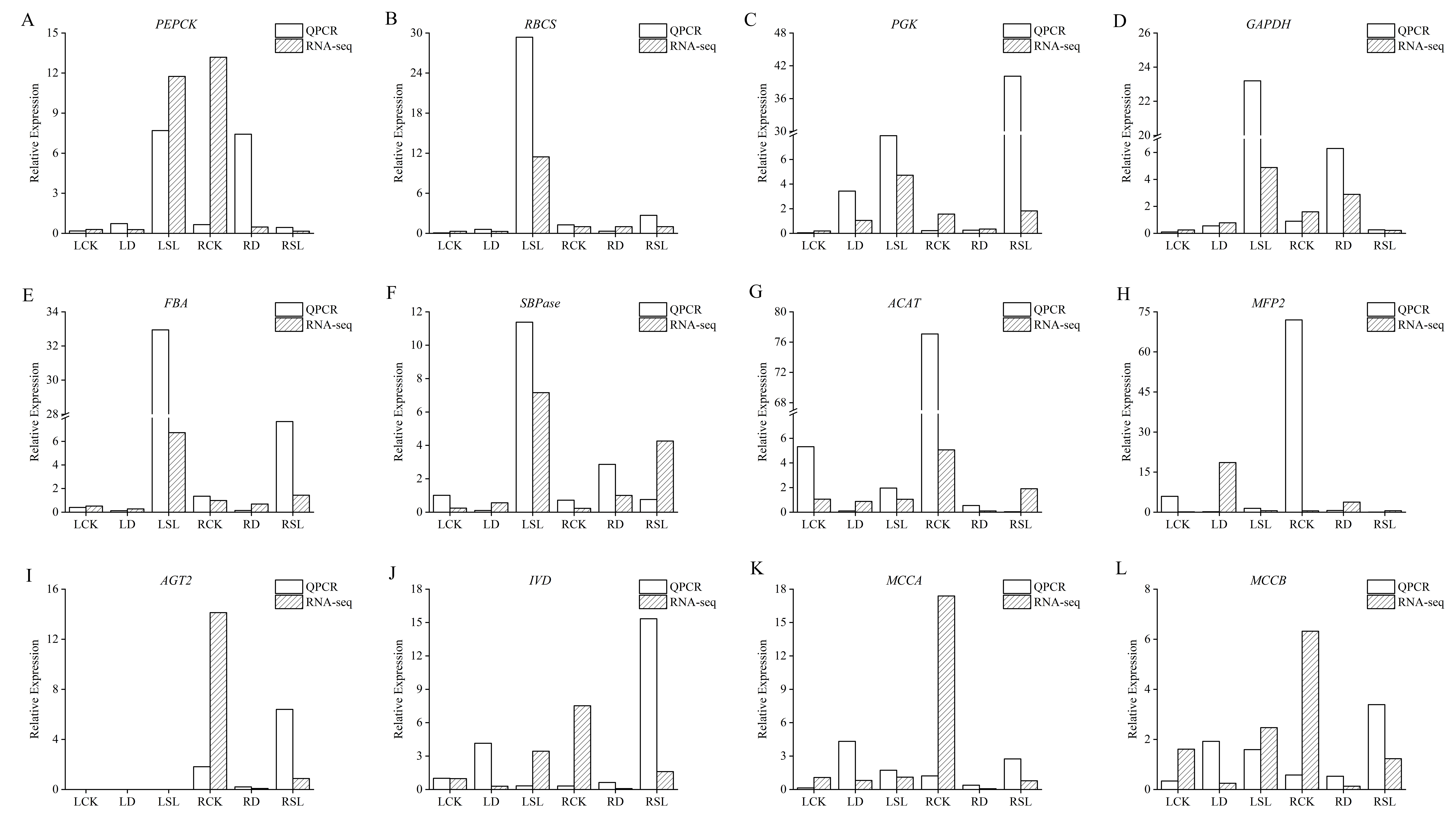

Supplement: Supplementary file 18 [file Image_6.tif]
